# Partner-inflicted brain injury may cognitively age survivors by over a decade

**DOI:** 10.64898/2026.09.24.26363984

**Published:** 2026-09-27

**Authors:** Craig W. McFarland, Eve M. Valera

**Affiliations:** 1 Department of Experimental Psychology, University of Oxford; Oxford OX2 6GG, UK; 2 Stanford Interdepartmental Neurosciences Program, Stanford University School of Medicine; Stanford, CA 94305, USA; 3 Department of Psychiatry, Mass General Brigham; Boston, MA 02129, USA; 4 Department of Psychiatry, Harvard Medical School; Boston, MA 02115, USA

## Abstract

**Introduction:**

More than a third of women in the United States are estimated to experience abuse by an intimate partner, and blows to the head and strangulation are common in such instances. Yet the cognitive consequences of partner-inflicted brain injuries remain poorly characterized relative to brain injuries from other mechanisms, such as athletics and military service, and study sample sizes have tended to be small. The degree to which these injuries accumulate into measurable cognitive harm, whether the harm of such injuries can be quantified in a meaningful way, and how that harm compares with better-recognized causes of repetitive head injury, has not been established.

**Rationale:**

We administered ten cognitive tests, scored across sixteen domains, to 207 women with lifetime exposure to physical intimate partner violence, of whom 67% (*n* = 139) reported at least one partner-inflicted brain injury. Since standardized scores are difficult to interpret clinically, we also expressed performance as cognitive age, the age at which the typical person in the general population performs equivalently. Finally, we compared women with brain injuries against women with no brain injury history from the same cohort, as this isolates injury from exposure to violence itself, since both groups experienced physical partner violence.

**Results:**

Women who had sustained any partner-inflicted brain injury performed below the general population mean on 11 of the 16 cognitive abilities measured, from reasoning (*d* = 0.32) to reaction time (*d* = 0.80), all *P* ≤ 0.011. Women reporting ten or more injuries demonstrated an overall cognitive function worse than nearly 80% of the general population (*d* = 0.82, *P* < 0.0001). Translating these deficits to cognitive age, women with ten or more brain injuries performed at a cognitive age more than two decades older than the general population on eight domains, the largest gaps being for simple attention (28.1 years, 95% CI [14.9, 41.4]; *d* = 0.78, *P* = 0.0002) and reaction time (27.8 years, 95% CI [18.1, 37.4]; *d* = 1.08, *P* < 0.0001). On psychomotor speed they also performed over a decade older than women exposed to physical intimate partner violence who had never sustained a brain injury (11.3 years, *P* = 0.033). After adjustment for eleven covariates, cognitive ability declined as injury count rose in six domains: psychomotor speed, reaction time, processing speed, motor speed, global cognition and verbal memory (partial *r* = −0.14 to −0.22, all *P* < 0.05), and 14 of the 16 coefficients were negative (two-sided sign test *P* = 0.004). We excluded scores flagged as invalid by the cognitive assessment per convention, yet observed that these flags fell disproportionately on the most impaired women, who scored 11 to 13 points lower (*d* = 0.60 to 0.84, all *P* < 0.001) on every domain the assessment did accept as valid.

**Conclusion:**

Women experiencing partner-inflicted brain injuries demonstrated cognitive aging of over 20 years, and yet, the true cognitive harms of such injury are likely more severe than those reported here. These results are particularly grave because partner-inflicted injuries may compromise the very cognitive capacities women must rely on to leave abusive relationships, care for their children, sustain employment, navigate legal proceedings, and remain safe post-violence. Taken together, these findings underscore partner-inflicted brain injury as a major, under-recognized source of cognitive harm, potentially affecting more women than brain injury in professional football or the military while lacking comparable clinical recognition and care.

## Summary Abstract

More than a third of women in the United States are estimated to experience abuse from an intimate partner, in which blows to the head, strangulation, and therefore brain injuries are common. Among 207 survivors of such physical violence, two-thirds reported a partner-inflicted brain injury. These women performed substantially worse than the general population across most cognitive measures, including memory and reaction time. Cognitive performance declined as injury count increased. Translated into cognitive age, survivors with the most partner-inflicted brain injuries performed over two decades older than the general population, and a decade older than survivors with no history of brain injury. Importantly, the cognitive assessment discards scores it judges unreliable, yet these came primarily from the most impaired women, suggesting that the true harm is likely greater than reported here. Such cognitive deficits may limit these women’s capacity to live to their potential, including keeping safe from abuse. These findings underscore partner-inflicted brain injury as a major, under-recognized source of cognitive harm, potentially affecting more women than brain injury in professional football or the military while lacking comparable clinical recognition and care.

## Introduction

More than a third of women in the United States are estimated to experience abuse from an intimate partner [1]. The threat of abuse intensifies in response to current events beyond their control, with rates reported rising during war [2,3], recessions [4,5], pandemic restrictions [6,7], floods [8,9], hurricanes [10–12], heatwaves [13,14], the Super Bowl [15], the World Cup [16], when the weekend comes [15,17], and, among other holidays, on Christmas [17].

Intimate partner violence (IPV) is self-evidently concerning, but a particularly troubling consequence is brain injury (BI). During abuse, survivors experience blows, kicks, falls, strangulations, and high-force impacts against walls or floors that can produce BIs [18–21]. The harms of BI compound, particularly as this “invisible trauma” can occur repeatedly without diagnosis, time to recover, or treatment for survivors of intimate partner violence [18,19]. The cognitive detriments of BI are frequently mistaken for psychological distress caused by the abuse, leaving the underlying BI unrecognized [18,20].

Heightened risks of stroke [22], seizures [23], and death [24] notwithstanding, BI impairs cognitive functions, often to debilitating extents [19,25–28]. Accordingly, billions of dollars have been invested in BI research among athletes and military personnel, informing legislation, clinical guidance, and safety initiatives [29–32]. Intimate partner violence has no comparable counterpart, although partner-inflicted BI is common, frequently repetitive, and may affect more people than BI in these traditionally studied populations [19,20,33]. Characterizing the breadth and severity of the cognitive harms from partner-inflicted BI is therefore crucial to understanding the impact of IPV, and consequently informing effective clinical care and relevant legislation.

Here we studied 207 women who had experienced physical violence from an intimate partner, with the number of reported partner-inflicted BIs ranging from none to 1,095. Each provided detailed accounts of their IPV exposure and completed ten cognitive tests, scored across sixteen cognitive domains. As cognition is shaped by many factors beyond BI, from years of education to substance use, we measured and accounted for these simultaneously [18,34,35]. This unusually deep characterization allowed us to test how cognition may be affected as partner-inflicted BIs accumulate. We emphasize that what follows concerns partner-inflicted BI and its measurable associations with cognition, not the intelligence or capability of the women who sustained it.

We expressed performance as cognitive age, defined here as the age at which the typical person in the general population performs equivalently. A 30-year-old performing like a typical 48-year-old therefore would have a cognitive age gap of 18 years. Intuitively, some women with severe BI histories were unable to complete particular cognitive tasks, produced scores deemed invalid by the assessment, or both. We therefore excluded their performance from relevant analyses per convention. As a result, the findings we report only capture the performance of survivors whose performance could be validly measured, and we bear in mind that the cognitive losses reported here are likely worse than what current cognitive methods can capture.

## Results

### Two-thirds of women reported at least one partner-inflicted brain injury

We began by quantifying partner-inflicted BI among the women. Two-thirds reported at least one injury (67%; *n* = 139) inflicted by their intimate partner. Over two-fifths (42%; *n* = 86) had multiple partner-inflicted BIs and 27% (*n* = 56) reported at least one BI from strangulation-related alterations in consciousness (Fig. 1 and Table S1). Among injured women, the median was two partner-inflicted BIs, one-quarter reported nine or more, and 15 reported more than 25, with counts reaching as high as 1,095. To limit the influence of extreme counts, we capped injury counts at 25 in our main analysis. This conservative choice did not drive the findings, which remained unchanged when women exceeding the cap were excluded (Table S5).

### Cognitive performance fell below population norms across most domains

To establish how these women performed relative to the general population, we next measured cognition across sixteen domains. Scores are age-adjusted, with a population mean of 100 and a standard deviation of 15. Mean scores were below the population mean on 14 of the 16 domains, 11 of which survived Bonferroni correction across sixteen tests (Fig. 2, white hatched bars, and Table S2). Women who had survived any physical violence demonstrated poorer reaction time (mean 88.9; *d* = 0.74; *P_bonf_* < 0.0001), simple attention (90.9; *d* = 0.61; *P_bonf_* < 0.0001), executive function (91.7; *d* = 0.55; *P_bonf_* < 0.0001), cognitive flexibility (92.0; *d* = 0.54; *P_bonf_* < 0.0001), composite memory (92.1; *d* = 0.53; *P_bonf_* = 0.53; *P* < 0.0001), verbal memory (92.6; *d* = 0.49; *P_bonf_* < 0.0001), motor speed (93.2; *d* = 0.45; *P_bonf_* < 0.0001), psychomotor speed (93.5; *d* = 0.44; *P* < 0.0001), global cognition (i.e., the composite measure of overall cognitive function; 93.8; *d* = 0.41; *P_bonf_* < 0.0001), visual memory (94.6; *d* = 0.36; *P_bonf_* < 0.0001) and reasoning (95.1; *d* = 0.32; *P_bonf_* = 0.0003).

We then investigated whether these cognitive deficits were more pronounced in women who reported any history of partner-inflicted BI. The same 14 cognitive domains were below the population mean, with considerably larger deficits among most of them. 11 observed cognitive deficits again remained significant after Bonferroni correction (Fig. 2, coloured bars; Table S2): Women who had sustained at least one partner-inflicted BI demonstrated poorer reaction time (88.0; *d* = 0.80; *P_bonf_* < 0.0001), simple attention (89.5; *d* = 0.70; *P_bonf_* < 0.0001), composite memory (91.2; *d* = 0.59; *P_bonf_* < 0.0001), verbal memory (91.5; *d* = 0.57; *P_bonf_* < 0.0001), cognitive flexibility (92.1; *d* = 0.53; *P_bonf_* < 0.0001), psychomotor speed (92.4; *d* = 0.51; *P_bonf_* = 0.0001), executive function (92.4; *d* = 0.51; *P_bonf_* < 0.0001), motor speed (92.5; *d* = 0.50; *P_bonf_* = 0.0001), global cognition (93.2; *d* = 0.45; *P_bonf_* < 0.0001), visual memory (94.2; *d* = 0.39; *P_bonf_* = 0.001) and reasoning (95.2; *d* = 0.32; *P_bonf_* = 0.011).

Deficits in complex attention and processing speed were smaller and did not survive correction, at 96.9 (*d* = 0.21; *P_bonf_* = 0.269; *P* = 0.017) and 97.7 (*d* = 0.15; *P_bonf_* = 0.620; *P* = 0.039) respectively across the cohort, and at 96.8 (*d* = 0.21; *P_bonf_* = 0.856; *P* = 0.054) and 96.5 (*d* = 0.24; *P_bonf_* = 0.075; *P* = 0.005) among the women reporting at least one partner-inflicted BI. Social acuity, the ability to identify emotional expressions, was indistinguishable from the population mean in both groups, at 99.8 (*d* = 0.01; *P_bonf_* = 1.00) and 99.1 (*d* = 0.06; *P_bonf_* = 1.00).

Mean working memory (102.9; *d* = 0.19; *P_bonf_* = 0.058) and sustained attention (104.2; *d* = 0.28; *P_bonf_* < 0.0001) were above the population mean across the cohort, and remained larger among the women reporting at least one partner-inflicted BI, at 101.9 (*d* = 0.13; *P_bonf_* = 1.00) and 103.5 (*d* = 0.24; *P_bonf_* = 0.015), respectively. Yet, working memory and sustained attention constituted the two domains with the most excluded scores, a point we return to in the discussion.

### Cognitive performance declines as partner-inflicted injuries accumulate

We next examined whether the number of partner-inflicted BIs a woman had sustained predicted her cognitive performance. To express the size of any gradient in interpretable terms, we grouped the women into four bands of injury burden (Methods; Fig. S1). Cognitive scores declined across all sixteen domains as partner-inflicted BI count increased (d = 0.06 to 0.70 from the lowest to the highest band; *P for trend* = 0.0033 to 0.65). The decline remained significant on overall cognitive function, reaction time, psychomotor speed and processing speed following false discovery rate correction (*d* = 0.55 to 0.70; all *q* = 0.032). Overall cognitive function, for instance, fell below the normative mean in each injury burden band, from 95.0 in women reporting none to 87.6 in those reporting ten or more (*d* = 0.30 to 0.82 against the normative mean, all *P* ≤ 0.035; *d* = 0.70 from lowest to highest band, *P* for trend = 0.0065; Fig. 3A, Fig. S2 and Table S3).

We then investigated whether these cognitive deficits were more pronounced still in women who reported ten or more partner-inflicted BIs. Deficits were largest in this group, extending to fourteen of the sixteen cognitive domains. Ten observed cognitive deficits remained significant after Bonferroni correction (Fig. 3A; Table S2), namely that women who had sustained ten or more partner-inflicted BIs demonstrated poorer reaction time (79.6; *d* = 1.36; *P_bonf_* = 0.0003), simple attention (85.7; *d* = 0.95; *P_bonf_* = 0.023), psychomotor speed (86.2; *d* = 0.92; *P_bonf_* = 0.002), composite memory (86.9; d = 0.88; *P_bonf_* = 0.018), executive function (87.2; *d* = 0.86; *P_bonf_* = 0.001), cognitive flexibility (87.3; *d* = 0.85; *P_bonf_* = 0.003), global cognition (87.6; *d* = 0.82; *P_bonf_* = 0.0001), verbal memory (87.8; *d* = 0.81; *P_bonf_* = 0.007), motor speed (88.0; *d* = 0.80; *P_bonf_* = 0.010) and processing speed (91.2; *d* = 0.59; *P_bonf_* = 0.016). Women in this greatest injury burden group scored on average 87.6 on overall cognitive function, therefore performing worse than nearly 80% of the general population (*d* = 0.82, P < 0.0001).

A deficit relative to population norms could reflect the conditions of living with a physically abusive partner, such as chronic stress or disrupted sleep, rather than brain injury. To mitigate this possibility, we compared women who had sustained a partner-inflicted BI against women in the same cohort who had endured physical partner violence with no history of partner-inflicted BI. Women reporting ten or more injuries scored below their uninjured counterparts on all sixteen domains (*d* = 0.06 to 0.70; *P* = 0.006 to 0.76; Table S3). The largest differences were on global cognition (*d* = 0.70; *P* = 0.007), reaction time (*d* = 0.64; *P* = 0.014), processing speed (*d* = 0.55; *P* = 0.006) and psychomotor speed (*d* = 0.55; *P* = 0.015), the same four domains the full-sample analysis above identified (Fig. 3A, Fig. S2 and Table S3).

Although we defined the most severe exposure group as ten or more BIs, this cut-off is one of many possible. To test whether our finding depended on our selected cut-off, we repeated the comparison at every threshold from one to twelve injuries (see Methods). A threshold of one injury separates the presence of injury from its absence rather than grading severity, and is treated separately below. From two injuries upward, which was the median number reported by injured women, cognitive scores were lower in the exposed group at every threshold on 13 of the sixteen cognitive domains (*d* = 0.01 to 0.73). For instance, psychomotor speed and processing speed were significantly lower at every one of those thresholds (*d* = 0.36 to 0.63; all *P* ≤ 0.032; Fig. 3C and Table S4). On global cognition, the deficit also worsened as the threshold rose, reaching significance from four upward and becoming nearly five times worse between one or more BIs (*d* = 0.15; *P* = 0.330) and twelve or more (*d* = 0.73; *P* = 0.011).

Finally, women who experience IPV often are assessed by whether they have a history of brain injury rather than by how many they have sustained [36,37]. To test whether injury presence alone was informative, we compared survivors reporting any partner-inflicted BI with those reporting none. The cognitive differences were small and imprecise (d = 0.01 to 0.26, all P ≥ 0.13; the largest, on working memory, 95% CI [−0.09, 0.61]), and therefore too uncertain to statistically separate a woman who has sustained a BI from one who has not.

Many factors beyond partner-inflicted BI shape cognition, including schooling, psychiatric distress, and injuries sustained before the relationship. We therefore adjusted every domain simultaneously for these potential confounders among eight others. After adjustment, injury count was associated with poorer performance on fourteen of the sixteen domains, including six meeting conventional levels of statistical significance at *P* < 0.05 versus 0.8 expected by chance (Table 1). The strongest was psychomotor speed (partial *r* = −0.22, *P* = 0.003), followed by reaction time, processing speed, motor speed, global cognition and verbal memory (partial *r* = −0.14 to −0.20, all *P* < 0.05). The consistency of direction across domains was itself unlikely under the null (two-sided sign test *P* = 0.004), though the domains share participants and a common exposure and so are not independent tests.

We recognized that asking women whose memory may have been impaired to recall how many injuries they sustained creates an inherent tension, since those with the most injuries might remember the fewest. We therefore tested whether memory predicted reported count. Women with poorer memory reported slightly more injuries, not fewer (composite memory *r* = −0.13, *P* = 0.07; verbal memory −0.14, *P* = 0.05), the opposite of what one might expect with forgetting or poor memory. Speed domains were associated with injury count more strongly than memory domains, the reverse of the pattern memory-driven under-reporting would produce (Table S10).

### Cumulative brain injury corresponds to more than a decade of additional cognitive aging

To convey the magnitude of these deficits in more intuitive terms, we translated the already observed standardized deficits into cognitive age, defined as the age at which the typical person performs at the same level. We derived cognitive age separately for each domain with a raw score that changes with age. Given that global cognition is an average of age-standardized scores across multiple cognitive domains, and therefore has no raw performance measure that changes with age, a cognitive age for global cognition cannot be calculated.

Women reporting ten or more partner-inflicted BIs performed over two decades older than the general population across eight cognitive domains (20.8 to 28.1 years; Table S12): simple attention (28.1 years; *P* = 0.0002), reaction time (27.8; *P* < 0.0001), verbal memory (26.0; *P* < 0.0001), motor speed (24.6; *P* < 0.0001), composite memory (22.7; *P* < 0.0001), cognitive flexibility (22.6; *P* < 0.0001), executive function (21.9; *P* < 0.0001) and psychomotor speed (20.8; *P* < 0.0001); see Fig. 4A for an illustrative example. Across the eleven domains significantly older than the general population, gaps ranged from 11.4 to 28.1 years.

Comparing women who had sustained ten or more partner-inflicted BIs against survivors who had sustained no BI, they performed over a decade older on four cognitive domains: simple attention (14.2 years; *d* = 0.40, *P* = 0.074), verbal memory (13.7; *d* = 0.44, *P* = 0.036), reaction time (11.9; *d* = 0.51, *P* = 0.036) and psychomotor speed (11.3; *d* = 0.51, *P* = 0.033). The difference reached significance on the last three of these and on processing speed (8.6 years; *d* = 0.52, *P* = 0.024; Table S12).

The gap widened as a woman sustained more partner-inflicted BIs (Fig. 4A and 4B). On psychomotor speed, for instance, even sustaining two or more partner-inflicted BIs equated to at least one decade, and potentially over two, of additional cognitive age relative to the general population (16.4 years; 95% CI [11.1, 21.8], *P* < 0.0001). The gap rose to 20.8 years in those reporting ten or more, across a gradient spanning the four exposure bands (*P* for trend = 0.0122). Treated as a continuous exposure rather than in bands, each log-unit increase in BI count corresponded to 4.58 additional years of cognitive age, adjusted for the same eleven covariates (SE 1.60, *P* = 0.005).

Calculating cognitive age can also produce negative values, meaning that some women performed better than expected for their chronological age (Fig. 4C). This is to be expected as roughly 50% of individuals should perform better than their predicted cognitive age within a normal population. Yet only about a third of survivors of physical IPV (35%, *n* = 68 of 197) performed better, whether or not they had sustained a partner-inflicted BI, falling to about one in five (22%, *n* = 7 of 32) among those who had sustained at least ten BIs from an intimate partner.

To determine whether the association depended on the specific analytic decisions we had made, we re-estimated it using alternative injury definitions, band schemes, outcome scaling, and covariate sets. The association held throughout (Tables S4 to S7). Finally, we asked whether the association reflected one mechanism of injury rather than cumulative injury itself, since blows to the head and strangulation injure the brain by different routes, the first by force and the second by depriving it of oxygen. The association was carried by blows to the head, although far fewer strangulation injuries were reported and the comparison is correspondingly less well powered to detect an effect of that mechanism (Table S11).

### Validity criteria exclude the most impaired women from the analysis

Every result reported above follows the standard practice of discarding cognitive scores that the assessment platform flags as invalid. To determine whether we were disproportionately excluding data from women with poorer cognitive performance, we compared the cognitive performance of women with at least one invalid score with those whose scores were never flagged.

The cognitive assessment platform flags a score as invalid when a response of a participant appears incompatible with genuine effort, and standard practice is to discard it. Forty percent of the women (40%; *n* = 82) in our study had at least one score flagged and accordingly excluded for the respective cognitive measures. In particular, women were consistently getting their scores flagged and excluded on two cognitive domains: their ability to stay focused on a task for an extended period of time (31%; *n* = 64) and their ability to hold information in their mind while performing a task, known as working memory (31%; *n* = 64; Fig. 5A).

When their flagged scores are excluded, survivors of physical partner violence appeared to have better sustained attention and working memory than the general population, scoring three to four points above the normative mean of 100 (*d* = 0.28 and 0.19; *P* < 0.0001 and *P* = 0.004). Yet, had we included the flagged scores in our original analysis, the survivors would have appeared to perform measurably worse than the general population (*d* = 0.48 and 0.36; *P* < 0.0001 and *P* = 0.0001) (Fig. 5B). When comparing women who had at least one flagged score with those who had none, on the domains the test did accept from them, such as their speed of thinking and their memory, those with a flagged score performed 11 to 13 points lower (Fig. 5C). In other words, the scores being excluded belong to the women with the most severe cognitive deficits in other domains.

## Discussion

Partner-inflicted BI is among the most common form of head trauma and among the least characterized in its impacts [18–20,38]. In the largest cognitively assessed cohort of survivors of physical IPV, women reporting ten or more injuries performed at a cognitive age more than two decades above the general population and over a decade above BI-free women also exposed to partner violence. Women with any BI performed significantly lower than the general population on a variety of cognitive measures. We consider what a deficit of this size means for survivors, why it may exceed established estimates, what our measurements miss, and what the findings imply for clinics and courts.

### A cognitive deficit of this size is consequential in ordinary life

We found that women who had suffered ten or more partner-inflicted BIs performed at a cognitive age over two decades older than the general population, and over a decade older than women who had endured physical IPV without sustaining a BI. In practical terms: the average 30-year-old woman who has sustained ten or more BIs from her partner performs cognitively on psychomotor speed as a 51-year-old woman in the general population, likely while having to navigate employment and childcare as well as possible legal demands, and continuing efforts to remain safe.

Performing cognitively at least a decade older than expected is demonstrably concerning. Notwithstanding how poorer cognitive ability is associated with earlier loss of independence [25], more motor vehicle crashes [39], and the ability to be employed [40], this result is particularly grave because partner-inflicted injuries may compromise the very cognitive capacities women must rely on to rebuild an independent life. These cognitive abilities, especially when significantly impaired, may compromise a survivor’s ability to leave abusive relationships, attain medication, care for their children, sustain employment, navigate legal proceedings, and remain safe post-violence.

Social acuity, the ability to distinguish emotional expressions, was the only cognitive domain in which performance was indistinguishable from the general population (*d* = 0.01, *P* = 1.00), and it did not decline detectably as injury burden rose (*r* = −0.05, *P* = 0.52). This relative preservation may be particularly meaningful in the context of an abusive relationship. Detecting subtle changes in a partner’s facial expression may allow a woman to recognize anger or agitation, anticipate escalating violence, and take steps to protect herself. Repeated exposure to interpersonal threat could therefore preserve, or potentially sharpen, attention to socially relevant emotional cues even as other cognitive abilities deteriorate as partner-inflicted BIs accumulate. We cannot determine whether this finding reflects adaptation to chronic threat or coincidence, but the cognitive capacity most relevant to detecting interpersonal threat was the cognitive that appeared to remain intact.

### Counting brain injuries helps detect cognitive harm

The present study demonstrates that cognitive deficit becomes more pronounced as partner-inflicted BIs accumulate. Women with any partner-inflicted BI did not differ significantly from women with none, while cognitive performance worsened with increasing injury burden. Prior studies point in a similar direction. Greater BI severity has been associated with poorer memory, learning, and cognitive flexibility [41], injury count with poorer visual memory [42], and repetitive head injury with greater cognitive symptom burden [43]. A recent study also found that learning and delayed-recall deficits were most apparent among women reporting more than six IPV-BIs [44]. Together, these findings suggest that cumulative exposure is important for understanding cognitive outcomes after partner-inflicted BI.

Our study extends this literature by showing that injury count is informative across a broad cognitive profile and remains so across multiple ways of defining exposure. This has a methodological implication for future studies. Classifying survivors only by the presence of BI may therefore obscure substantial variation in cumulative exposure and weaken associations with cognition. Alongside evidence that brain injury is often repetitive in IPV [45], our findings suggest that injury count should be treated as a core dimension of exposure to better capture variation in cognitive outcomes.

### A well-studied deficit in an understudied population

BI in sports and the military provides the firmest available benchmark against which to place the present findings. Repeated head injury in athletes and veterans has been studied extensively, attracting billions of dollars in funding and dedicated federal programs [32], and more than four hundred articles on football concussion in the New York Times between 2007 and 2013 alone [38].

Placing these deficits in context is difficult, because effect estimates drawn from different batteries, reference groups, exposure definitions and intervals since injury are not strictly comparable. Provided this caveat, meta-analyses report cognitive deficits of 0.29–0.49 standard deviations after sports concussion and 0.12–0.33 after blast-related mild traumatic BI [46–48], with poorer performance also observed after repetitive subconcussive exposure and recurrent concussion [49,50]. Here, women reporting partner-inflicted BI showed deficits of 0.45 standard deviations in global cognition, 0.51 in psychomotor speed, 0.59 in memory and 0.80 in reaction time. These estimates sit at or above those reported in sports and military populations. Similarly, a 30-year study associated two or more head injuries with cognitive decline equivalent to 9.7 additional years of age, compared with the 16.4-year cross-sectional cognitive-age gap observed here [51].

Importantly, prior sports literature has characterized athletes with three or more prior concussions as a highly exposed group [52], whereas here, the median injured woman reported two BIs, a quarter reported nine or more, and counts reached 1,095. In our highest band of ten or more BIs, the deficit reaches 0.82 standard deviations below the population and 20.8 years of cognitive aging. No published benchmark exists at that level of exposure, and we report these estimates to describe a range that has not previously been documented.

A variety of factors may explain the comparatively large cognitive deficits observed here, including the circumstances surrounding each BI. Athletes, for instance, wear protective equipment manufactured to standardised impact criteria [53]. When an injury is suspected, they are removed from play, examined by trained staff, and given a defined interval before returning [54]. Even there, quantifying cumulative head-impact exposure remains methodologically unresolved [55]. These protections did not extend to the present cohort, as seldom do IPV survivors receive immediate assessment [41], have their injuries monitored [20,56], have any opportunity to remove themselves from the setting in which the injury occurred, likely recover before another injury is sustained [18,19], or wear protective headgear at the time of partner abuse. Help-seeking following BI is itself additionally constrained by stigma, unmet service needs, and mandatory reporting requirements [57–59].

### The true cognitive harm is likely larger

We anticipate that the real cognitive harms of partner-inflicted BI are worse than reported here. The women who entered were safe enough to be contacted, had time and privacy to participate, and retained sufficient cognitive capacity to complete detailed interviews, cognitive testing, and be coherent enough to communicate. The cohort was also unusually educated with 62% holding a college degree against 40.1% of women aged 25 and over nationally [60]. Because higher education predicts stronger cognitive performance and may itself protect against partner violence [61,62], this sample was both less representative of women at greatest risk and more likely to score above population norms.

In addition, among the women for whom the timing of injury was recorded, the most recent partner-inflicted BI preceded testing by a median of 7.5 years, and 94% had been injured more than a year earlier. Cognitive performance can improve after BI, particularly in the first year following severe injury [63], so testing years later may capture the impairment remaining after recovery rather than its full initial severity. Women still living under immediate threat, or whose injuries left them unable to complete a research visit, are less likely to participate in our studies, as well as women residing in shelters.

The most severe form of bias here is survival itself. By definition, this study could include only women who lived following their BI. Put another way, those most likely to have been cognitively affected by partner-inflicted BIs were also the most likely to have been unable to participate in our study or to not have survived.

Our analysis choices were also conservative in ways that likely understate the harm. We capped injury counts at 25 even though the highest reported count was 1,095, excluded scores flagged by the cognitive battery [64–69] even though they belonged to women who performed worse on accepted domains, and restricted cognitive ages to 18 to 90 years, meaning that performance below the oldest reference group could not make the estimated gap any larger. Each decision compressed the most severe exposure or impairment, making the cognitive harms observed here more likely to be underestimated than overstated.

### Interpretation and limits

As a cross-sectional study, we have no pre-injury cognitive baseline. We therefore cannot establish that partner-inflicted brain injuries caused the observed deficits, nor rule out that the women who went on to sustain multiple injuries were already performing poorly on cognitive testing. Yet, we have strong reason to assume causality. In other populations, head injury has been shown to precede cognitive decline rather than follow it in a community cohort followed for thirty years [51]. Moreover, cumulative head-impact exposure across a career in contact sport is associated with later cognitive impairments [70], and with chronic traumatic encephalopathy at autopsy, whose severity tracks dementia diagnosed before death [71,72]. These designs, however, cannot establish temporal order. Encouragingly, efforts to quantify the long-term cognitive risk carried by IPV are beginning [73].

In addition, cognition was measured once, with a single battery, and the highest-exposure bands contain relatively few women, which is reflected in the width of their confidence intervals. It also remains an open question how to quantify a survivor’s cognitive impairment when the deficit is so severe that the survivor cannot complete the cognitive assessment task itself [74]. Finally, we translated cognitive performance to cognitive age so that the finding could be interpreted by a clinician, a court, or a legislator. However, we acknowledge that a difference in years describes how a younger woman resembles an older one and does not establish that her brain itself has aged.

### Making an invisible trauma visible in clinics and courts

Screening for partner violence typically assesses physical injury and psychological distress, but rarely cognition [20]. In the context of deficits equivalent to more than a decade of cognitive aging, omitting cognition risks missing a potentially major consequence of partner-inflicted BI. Screening for BI count is therefore vital for better capturing the cognitive harms of IPV.

Detection matters because effective cognitive treatment exists. Cognitive rehabilitation exists that targets memory, attention, processing speed, and executive function, while occupational and speech-language therapies help women apply compensatory strategies [75]. Cognitive behavioral therapy adapted for BI can also treat co-occurring anxiety and depression when delivered with manuals tailored to BI victims [76]. These treatments should be routinely integrated into care for partner violence. Likewise, in sports and military medicine, an identified BI initiates established pathways for monitoring, rehabilitation, and graded return to activity [54,77]. Women injured by intimate partners would benefit from the same continuity of care, particularly as BI does not invariably heal steadily, and some survivors deteriorate years later [78]. A single cognitive assessment or treatment should therefore not be followed by an assumption of recovery.

Of serious importance are the legal implications of severe cognitive deficits. Our findings suggest that BI, especially when repeated, can severely affect the very capacities on which legal judgments depend. The impaired memory observed here may make the chronology of repeated violence less precise, while slowed processing and executive dysfunction commonplace in our cohort may impede testimony, paperwork, and compliance with legal procedure. If these consequences are interpreted as dishonesty, instability, or noncompliance, a woman can be punished for the injury her partner caused. Courts and disability evaluators should not automatically discard cognitive scores flagged as unreliable. Here, women with flagged scores performed worse on measures the same battery accepted, indicating a need for fuller evaluation rather than erasure from the record. BI-informed assessments and procedural accommodations are therefore necessary in disability, criminal, and custody proceedings, particularly where cognitive impairment might otherwise be used to question a woman’s credibility or fitness to be a custodial parent [56].

We note that translating newly established harms of partner violence into legal recognition has clear precedent. After non-fatal strangulation was linked to roughly six- to sevenfold higher odds of attempted and completed intimate partner homicide [79], federal law made strangulation of an intimate partner a felony within federal jurisdiction in 2013 [80], and by 2023 all 50 states had criminalised non-fatal strangulation as a felony, Ohio being the last to do so [81]. The substantial cognitive harms documented here strongly suggest a comparable legal response, with the law recognizing repeated partner-inflicted BI as a grave and cumulative consequence of IPV.

The brain injuries endured by survivors, and their cognitive harms, often remain invisible because clinics and courts rarely look for them. Here, we offer a metric of cognitive age that quantifies one of the many serious harms of partner-inflicted violence. In doing so, we hope to advance meaningful efforts to measure the harms survivors have immeasurably endured.

## Supplementary Material

1

## Figures and Tables

**Fig. 1.**
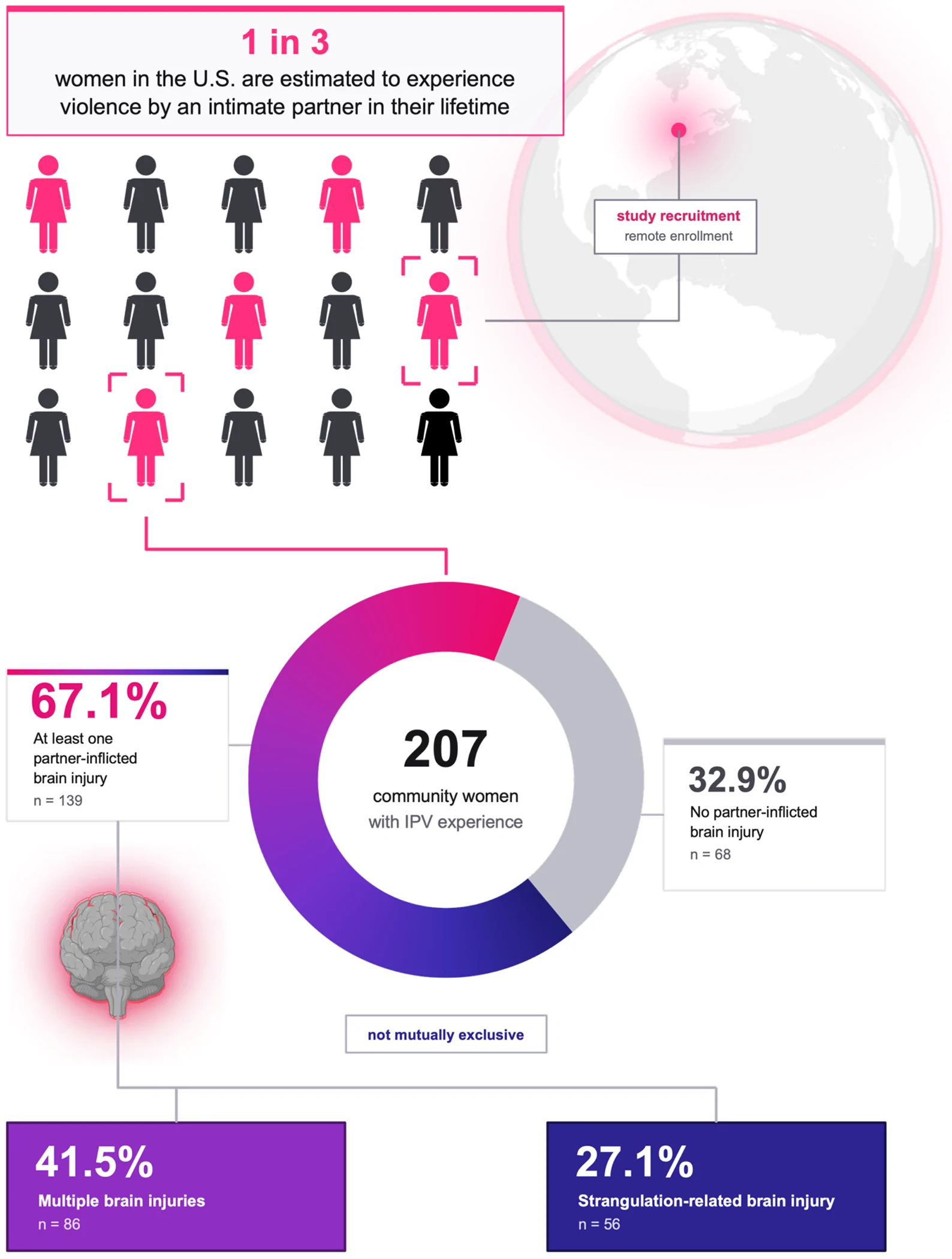
Partner-inflicted brain injury in a community sample of women exposed to intimate partner violence. Over one in three women in the United States are estimated to have experience abuse from an intimate partner in their lifetime. Among the 207 women studied here, all reporting lifetime exposure to partner violence, 139 (67.1%) reported at least one partner-inflicted BI and 68 (32.9%) reported none. Among those who had sustained BIs, 86 women (41.5% of the sample) sustained more than one and 56 (27.1%) sustained a strangulation-related injury; the two categories overlap.

**Fig. 2.**
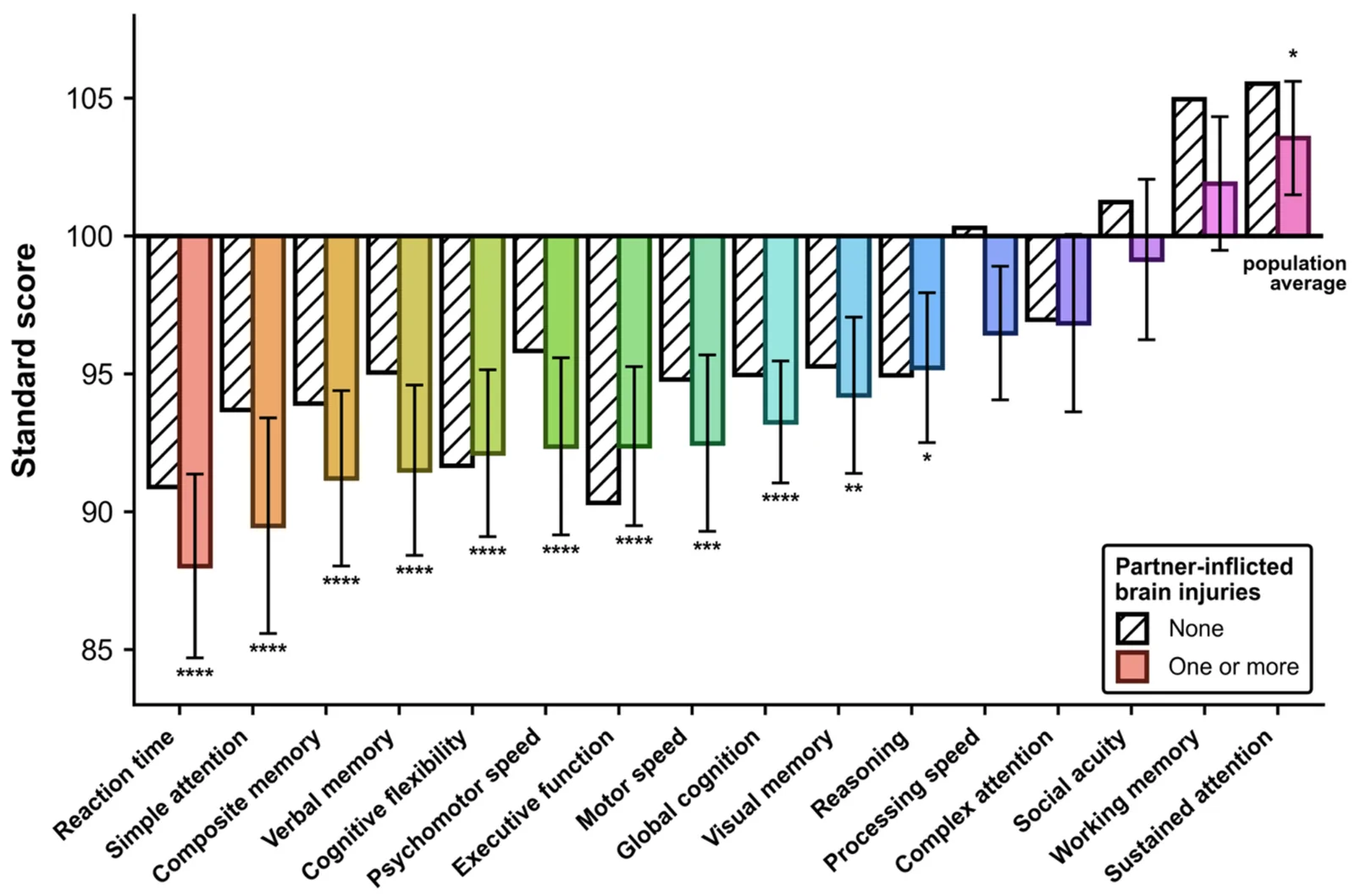
Cognitive performance against population norms in women with and without partner-inflicted brain injury. Bars give the mean standard score for each domain in the 68 women reporting no partner-inflicted brain injury (white, hatched) and the 139 women reporting at least one (coloured). Both groups experienced physical violence from an intimate partner, so the contrast is injury rather than exposure to violence. Domains are ordered by the injured group, lowest to highest, and the horizontal line marks the population average of 100. Error bars are 95% confidence intervals for the injured group. Asterisks denote significance for the injured group against the population average of 100, after Bonferroni correction across sixteen tests: *P < 0.05; **P < 0.01; ***P < 0.001; ****P < 0.0001. The uninjured group is shown for reference only. No significance is marked for it, and no test between the two groups is displayed. However, mean scores fell significantly below the population average on four domains in the uninjured group and significantly below the population average on eleven domains among injured women. Social acuity sits at the population average in both.

**Fig. 3.**
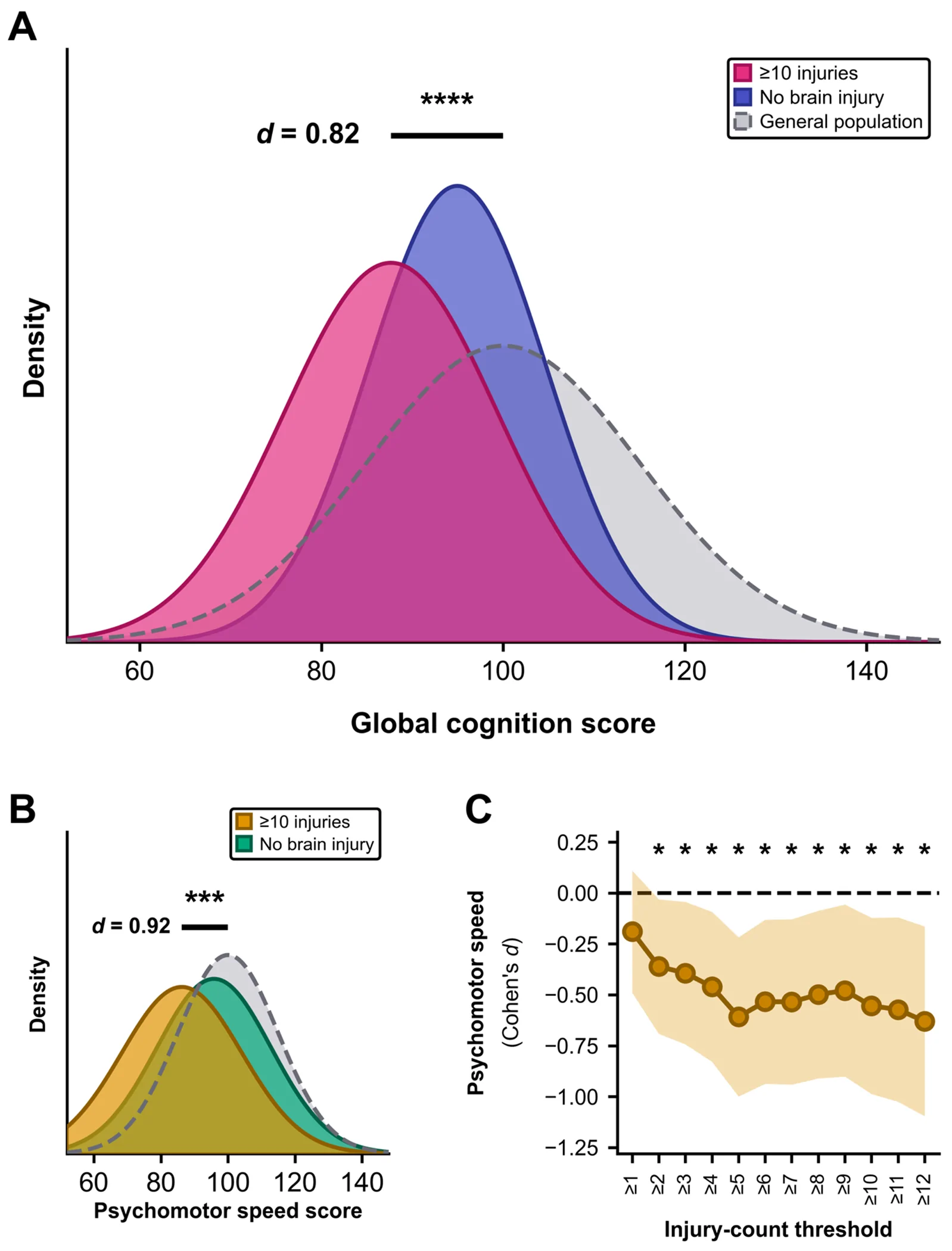
The deficit scales with injury count and is independent of the threshold chosen. **(A)** Global cognition drawn as normal distributions with a common standard deviation of 15, for the general population (grey, dashed, mean 100), women reporting no partner-inflicted BI (indigo, 95.0) and women reporting ten or more (pink, 87.6). The bar marks the difference between the heavily injured group and the population mean, 0.82 SD, *P_bonf_* = 0.0001); the same group sits 0.70 SD below uninjured women in this cohort (d = 0.70, P = 0.007). **(B)** The same construction for psychomotor speed, with women reporting no partner-inflicted BI (green, 95.8) and ten or more (orange, 86.2) against the general population (grey, dashed). The bar marks 0.92 SD below the population mean, *P_bonf_* = 0.002); the within-cohort difference is 0.55 SD (P = 0.015). Asterisks in (A) and (B) denote unadjusted one-sample tests against the population mean (*P < 0.05; **P < 0.01; ***P < 0.001; ****P < 0.0001); the Bonferroni-corrected values are those quoted above and in Table S2. **(C)** The within-cohort contrast on psychomotor speed re-estimated at twelve injury thresholds, each comparing women reporting at least that many injuries against women reporting none. Negative values indicate poorer performance in the exposed group, shading gives the 95% confidence interval, and asterisks mark P < 0.05. Every threshold from two injuries upward yields a significant difference (*d* = 0.36 to 0.63; Table S4); two is the median number of injuries reported by injured women. All sixteen domains are ranked by effect size in Fig. S2.

**Fig. 4.**
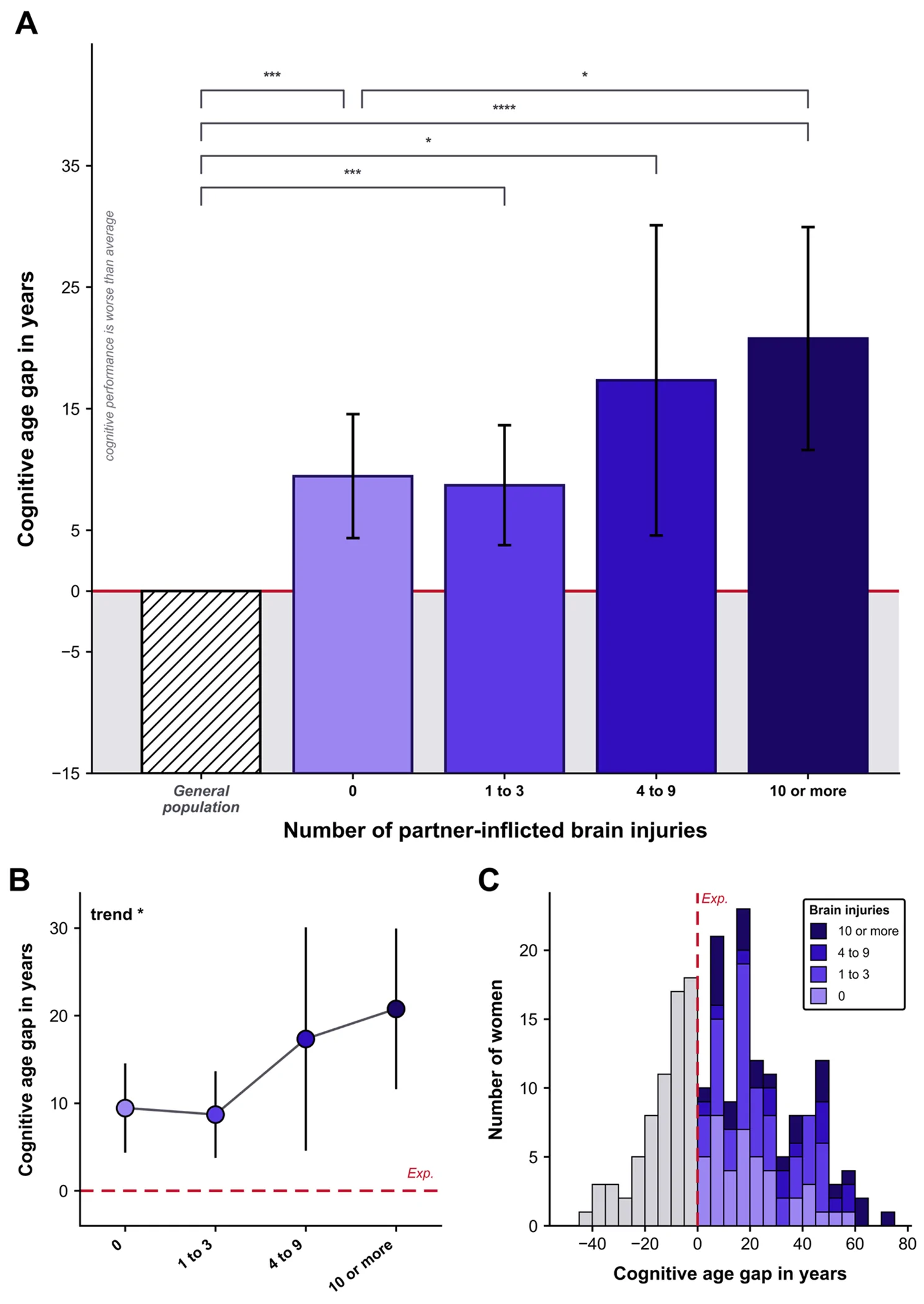
Cognitive age gap by cumulative partner-inflicted brain injury. **(A)** Mean cognitive age gap in years, derived from psychomotor speed, for each exposure band, against a general-population reference of zero (hatched bar). Women reporting no partner-inflicted brain injury sat 9.5 years above the reference, against 8.7 years for one to three injuries, 17.3 for four to nine, and 20.8 for ten or more (*n* = 63, 80, 22 and 32 with a computable gap). Error bars are 95% confidence intervals. Brackets give tests against the population reference, and the lowest pair the within-cohort comparison between women reporting none and women reporting ten or more, 11.3 years (*P* = 0.033). Shading below zero denotes performance better than the population average for age. **(B)** Band means with 95% confidence intervals across the four ordered bands (*P* for trend = 0.0122). **(C)** Distribution of individual cognitive age gaps, stacked by exposure band. Bars left of the dashed line denote women performing at a cognitive age younger than their own. The proportion performing better than expected for their age fell from 35% among women reporting no injury to 22% among those reporting ten or more, against 50% expected in the normative sample. Asterisks: *P < 0.05; **P < 0.01; ***P < 0.001; ****P < 0.0001.

**Fig. 5.**
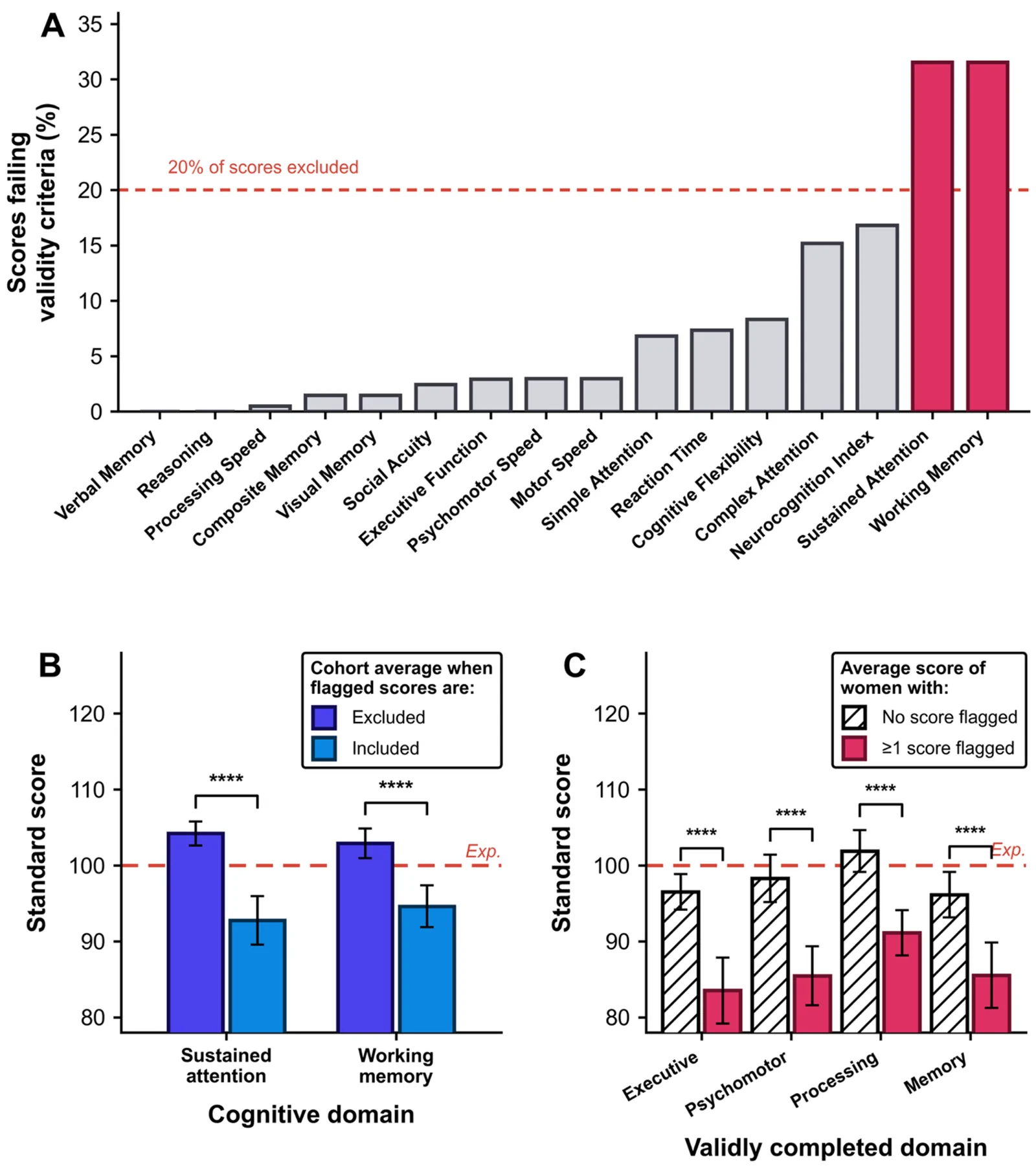
Excluding test scores that fail validity criteria removes the most impaired women. Scores flagged by the cognitive battery’s embedded validity indicator are excluded from all analyses reported here. Scores are flagged as potentially unreliable based on response speed, error patterns, or other indicators of lack of genuine effort. **(A)** Percentage of scores flagged, by domain. Sustained attention and working memory, both scored from the four-part continuous performance test, are flagged in 31% of scores each (pink); the dashed line marks 20%. **(B)** Cohort average on those two domains under two conventions, with flagged scores excluded (indigo) or included (blue). Excluding them places both domains above the population average of 100; including them places both below. **(C)** Average score of women who had at least one score flagged elsewhere in the battery (pink) against women who had none (hatched), shown only for domains the battery accepted from both groups. Women with a flagged score scored 11 to 13 points lower on the domains they did complete validly. Bars give 95% confidence intervals. Asterisks: *P < 0.05; **P < 0.01; ***P < 0.001; ****P < 0.0001.

**Table 1.** Adjusted associations between cumulative partner-inflicted brain injury and cognitive performance. Multivariable linear regression of standard score on log injury count, adjusted for age, education, race, non-partner adult and childhood brain injury, post-traumatic stress, depression, anxiety, alcohol use, childhood maltreatment, and abuse severity. *B* is the unstandardized coefficient with its standard error, giving the change in standard score per unit increase in log injury count, and *P* the two-sided probability. All sixteen domains are reported, from a single specification applied to each in turn. Six associations reach *P* < 0.05, against 0.8 expected were no association present, and fourteen of the sixteen coefficients are negative (two-sided sign test *P* = 0.004). Under Benjamini-Hochberg control across the sixteen domains, four remain significant at *q* < 0.05 (psychomotor speed, reaction time, processing speed and motor speed).

Domain	*n*	*B (SE)*	*Partial r*	*95% CI*	*P*
Psychomotor speed	196	−3.89 (1.27)	−0.22	−0.35 to −0.08	0.0025
Reaction time	189	−3.60 (1.34)	−0.20	−0.34 to −0.05	0.0081
Processing speed	203	−2.81 (1.07)	−0.19	−0.32 to −0.05	0.0093
Motor speed	196	−3.17 (1.25)	−0.18	−0.32 to −0.04	0.0121
Global cognition	168	−1.93 (0.88)	−0.17	−0.32 to −0.02	0.0292
Verbal memory	203	−2.58 (1.30)	−0.14	−0.28 to −0.00	0.0487
Composite memory	200	−2.53 (1.33)	−0.14	−0.28 to 0.01	0.0588
Visual memory	200	−1.70 (1.20)	−0.10	−0.24 to 0.04	0.1560
Simple attention	191	−2.23 (1.63)	−0.10	−0.24 to 0.04	0.1730
Social acuity	200	−0.58 (1.23)	−0.03	−0.18 to 0.11	0.6393
Working memory	142	−0.25 (1.03)	−0.02	−0.19 to 0.15	0.8088
Sustained attention	142	−0.17 (0.80)	−0.02	−0.19 to 0.15	0.8374
Executive function	198	+0.12 (1.20)	+0.01	−0.14 to 0.15	0.9214
Complex attention	173	−0.08 (1.38)	−0.00	−0.16 to 0.15	0.9556
Reasoning	205	+0.05 (1.11)	+0.00	−0.14 to 0.14	0.9656
Cognitive flexibility	187	−0.05 (1.24)	−0.00	−0.15 to 0.14	0.9681

## Data Availability

Direct identifiers have been removed from the analysis dataset. The de-identified data supporting this study are available from the corresponding author upon reasonable request. All data will be uploaded to the Federal Interagency Traumatic Brain Injury Research (FITBIR) data repository after completion of the study, at https://fitbir.nih.gov/
