# Supplementary material for: Partner-inflicted brain injury may cognitively age survivors by over a decade
Source: medRxiv. 2026 Sep 27:2026.09.24.26363984. Preprint. [Version 1] doi: 10.64898/2026.09.24.26363984 (PMC13622419; doi:10.64898/2026.09.24.26363984)
Supplement: Supplementary file 1 [file NIHPP2026.09.24.26363984v1-supplement-1.pdf]

medRxiv preprint doi: https://doi.org/10.64898/2026.09.24.26363984; this version posted September 27, 2026. The copyright holder for this preprint (which was not certified by peer review) is the author/funder, who has granted medRxiv a license to display the preprint in perpetuity.

It is made available under a CC-BY 4.0 International license .

Supplementary Materials for

Partner-inflicted brain injury may cognitively age survivors by over a decade

Craig W. McFarland, Eve M. Valera

Corresponding author. Email: craig.mcfarland@psy.ox.ac.uk

This PDF file includes:

Materials and Methods
Supplementary Text
Figs. S1 and S2
Tables S1 to S12
Data S1

medRxiv preprint doi: https://doi.org/10.64898/2026.09.24.26363984; this version posted September 27, 2026. The copyright holder for this preprint (which was not certified by peer review) is the author/funder, who has granted medRxiv a license to display the preprint in perpetuity.

It is made available under a CC-BY 4.0 International license .

Materials and Methods

Experimental design

This was a cross-sectional study of cognitive functioning in community-dwelling women with lifetime exposure to physical intimate partner violence. The study was designed to test whether cognitive performance declines as partner-inflicted injuries accumulate.

Participants and recruitment

Data collection took place between 2020 and 2026 as part of a larger ongoing study of partner-violence-related brain injury. Participants were recruited through flyers distributed by community partners, an institutional online research platform, and social media. Community partners included hospital-based programs, police-department-affiliated initiatives, and community organizations focused on violence prevention and survivor support. To be eligible, women had to have experienced at least one instance of physical partner violence and be at least 18 years old. A personal history of brain injury was not required. Of 210 eligible women with both brain injury and cognitive data, three were later excluded: two for pre-existing neurological conditions requiring neurosurgery, and one for inconsistent self-report across instruments, leaving 207. All processes and procedures were approved by our hospital's Institutional Review Board (IRB), and every participant provided informed consent.

Procedure

Sessions were conducted by video conference and completed in a single session. Participants were instructed to find a private, distraction-free location. Those without a personal computer were offered a study device. Following informed consent, demographic information was collected by interview. Participants then completed the computerized cognitive battery independently before the abuse and injury interviews. Study staff offered participants the opportunity to prepare a safety plan in advance, including strategies for discreetly pausing the session. Participants could take breaks or withdraw at any point and received a gift card on completion.

Exposure assessment

Brain injury exposure was assessed with two structured interviews: the Brain Injury Severity Assessment (BISA) and the Ohio State University Traumatic Brain Injury Identification Method (OSU TBI-ID). The BISA applies the criteria of the American Congress of Rehabilitation Medicine [82] to anything a partner did to the woman, defining a brain injury as a traumatically induced physiological disruption of brain function manifested by loss of consciousness, loss of memory for events immediately before or after the event, alteration of mental state at the time, or focal neurological deficit. The OSU TBI-ID documents lifetime injuries from any cause and asks about loss of consciousness, being dazed, or a gap in memory. A brain injury identified by either interview was counted. Following this definition and prior work in this cohort [42,83–85], alterations in consciousness resulting from violent shaking and from strangulation-induced anoxia or hypoxia were also classified as brain injuries.

Where a participant reported an alteration in consciousness following an incident with a partner, follow-up questions established the mechanism. Events attributed to strangulation or choking were classified as anoxic; events attributed to traumatic force to the head were classified as blunt-force trauma. Strangulation or shaking events that did not produce an alteration in consciousness were not recorded.

medRxiv preprint doi: https://doi.org/10.64898/2026.09.24.26363984; this version posted September 27, 2026. The copyright holder for this preprint (which was not certified by peer review) is the author/funder, who has granted medRxiv a license to display the preprint in perpetuity.
It is made available under a CC-BY 4.0 International license .

The primary exposure was the total count of partner-inflicted brain injuries: blunt-force, anoxic, the two combined categories, violent shaking, and moderate-to-severe events. Counts were capped at 25, beyond which an accurate estimate is difficult to obtain, following prior work in this cohort [42]. Fifteen women, 11% of those exposed, exceeded the cap, with counts up to 1,095. Capping treats these women identically to a woman reporting 25 events and is therefore conservative. A mild-only definition excluding moderate-to-severe events is reported as a sensitivity analysis (Table S7). Counts were log-transformed as log(1 + count) for continuous analyses. Injuries sustained outside the relationship, in adulthood and in childhood, were documented separately and retained as covariates.

Cognitive assessment

Cognition was measured with CNS Vital Signs, a computerized battery yielding age-adjusted standard scores with a population mean of 100 and standard deviation of 15 across the sixteen domains reported by the version of the battery administered here [86]. Domain names follow the battery's own reporting, with one exception. The domain reported here as global cognition is the battery's Neurocognition Index, the average of its five core domain scores: composite memory, psychomotor speed, reaction time, complex attention and cognitive flexibility. The battery was chosen because it offers a comprehensive assessment of cognitive performance while remaining accessible to a population that is difficult to reach. Administration and scoring are automated, so performance does not depend on rater judgement, which matters where an examiner aware of a woman's abuse history might otherwise influence testing. Its subtests are computerized adaptations of conventional neuropsychological tests, including the Stroop, Shifting Attention, Continuous Performance and Symbol Digit Coding tasks (Table S9), and concurrent validity against their standard forms has been established [86]. It can be delivered without a laboratory visit, which allows assessment of women for whom in-person testing is impractical or unsafe. All cognitive assessments were administered remotely, in a private location of the participant's choosing, with a member of the study team available by video for the duration. The normative sample for this battery was tested under supervised administration. The battery reports raw scores alongside age-adjusted standard scores, so the normative relation between age and raw performance can be estimated directly from the data. The battery applies an embedded validity indicator to each domain and flags scores it judges unreliable [64], and the consequences of deleting those scores are examined in the main text and in supplementary text.

Derivation of cognitive age

Cognitive aging was estimated by calculating how many years older the average woman in the general population would need to be to show the same performance on a given measure. Because the battery reports both raw and age-adjusted standard scores, the two coefficients needed for this conversion can be estimated by regressing raw score on age and standard score. The age coefficient gives the normative change in raw score per year of age, and the standard-score coefficient gives the change in raw score per standard-score point. Their ratio gives the number of years of normative aging equivalent to one standard-score point. A woman's cognitive age gap is that ratio multiplied by her own departure from the standard-score mean of 100, and her cognitive age is her chronological age plus that gap. A gap of zero denotes performance exactly as expected for her age, a positive gap performance typical of an older woman, and a negative gap that of a younger one. As speed measures decline more steeply with age than memory measures, a given

medRxiv preprint doi: https://doi.org/10.64898/2026.09.24.26363984; this version posted September 27, 2026. The copyright holder for this preprint (which was not certified by peer review) is the author/funder, who has granted medRxiv a license to display the preprint in perpetuity.
It is made available under a CC-BY 4.0 International license .

standardized decrement converts to more years on speed domains, and the years metric is therefore reported throughout alongside the underlying standardized effect.

Importantly, cognitive age is an interpretive rescaling of the observed cognitive-performance difference as opposed to an independent outcome. All inferences regarding significantly poorer cognition are therefore evident in the original age-adjusted standard scores and do not depend on the age-equivalent transformation. Cognitive age is also meaningful only within the age range the normative data describe. A woman scoring far below the mean would otherwise be assigned a cognitive age below zero, and one scoring far above it an age beyond any human lifespan. These values would mean only that performance fell outside what the reference data can represent, and we therefore constrained every estimate to the 18 to 90 years range that the normative data cover. Since the bounds are defined relative to a woman's own age, the lower bound binds younger women more tightly, and the uninjured band is the youngest in the cohort. Bounding therefore raises the mean in that band and lowers it in the most exposed one. Before bounding, mean gaps across the four bands were 7.4, 7.8, 18.7 and 24.4 years, and after bounding they are 9.5, 8.7, 17.3 and 20.8 years. Bounding compresses the gradient, so the reported estimates are conservative. Separately, derived age metrics can carry a regression artifact whereby the estimated gap correlates with chronological age, as described for brain-age estimates [87]. We tested for this directly and found none: the cognitive age gap was not associated with chronological age ($r = -0.08$, $P = 0.27$), as expected when scores are age-adjusted before conversion.

In addition, domains differ in how steeply raw performance declines with age, so the same standard-score deficit converts to more years in some domains than others. Certain cognitive skills, such as simple attention, are largely preserved across the lifespan and are not expected to change much as one ages. The slope relating raw performance to age in these domains is therefore relatively flat, and because the conversion divides by that slope, a given deficit translates into an exceedingly large number of years. Large deficits on these cognitive domains consequently yield substantial cognitive age gaps. We therefore interpret the larger estimates as indicating that performance falls outside the range the normative data describe, rather than as precise age equivalences. As noted above, we bound these estimates to the 18-to-90 year range the normative data cover, which truncates the largest gaps from above and therefore makes the reported values conservative. However, we acknowledge that it remains difficult to equate a severe impairment of an otherwise age-stable ability like simple attention to a numerical age, and the domains whose performance declines steadily with age, such as psychomotor speed and processing speed, therefore yield the most interpretable conversions.

Exposure bands. Participants were classified as reporting no partner-inflicted brain injury (BI), 1–3 injuries, 4–9 injuries, or ≥10 injuries. These cut-points were selected for clinical interpretability and to ensure adequate precision within each group, with at least 23 participants in every band. To evaluate whether the findings depended on this particular categorization, we examined all 390 alternative four-band schemes formed from candidate cut-points between 1 and 25 injuries that placed at least 15 participants in every band. The cognitive-age gradient was statistically distinguishable from zero in 96% of these schemes, with P values ranging from .001 to .072 (median, .011). The prespecified scheme ranked 205th of 390 by effect size, placing it in the weaker half of the candidate schemes and confirming that it did not maximise the observed association. We further conducted a permutation test in which the best-fitting banding scheme was reselected within each of 5,000 permutations. This analysis accounted for the full set of alternative cut-points and continued to support the cognitive-age gradient (P = .006). Although several

medRxiv preprint doi: https://doi.org/10.64898/2026.09.24.26363984; this version posted September 27, 2026. The copyright holder for this preprint (which was not certified by peer review) is the author/funder, who has granted medRxiv a license to display the preprint in perpetuity.

It is made available under a CC-BY 4.0 International license .

alternative schemes produced marginally larger effect estimates, they created narrower middle-exposure groups and less stable, non-monotonic estimates. Finally, abandoning bands altogether, we re-estimated the contrast at each of twelve injury thresholds. Effect sizes rose from d = 0.19 at one or more injuries to d = 0.63 at twelve or more, and eleven of the twelve thresholds yielded a difference distinguishable from zero (Table S4).

Covariates

Each model adjusted for the same eleven covariates selected for prior association with both injury exposure and cognitive performance: age, education, race, BIs sustained outside the relationship in adulthood, BIs sustained in childhood, post-traumatic stress (PCL-5) [88], depression (PHQ-9) [89], anxiety (GAD-7) [90], alcohol use (AUDIT) [91], childhood maltreatment (Childhood Trauma Questionnaire [92], and abuse severity over the past twelve months (Composite Abuse Scale) [93,94]. Abuse severity was indexed by the frequency of abusive behaviors over the past twelve months, the interval the Composite Abuse Scale is designed to assess: the concern this covariate addresses is that ongoing abuse rather than past injury depresses test performance, so the relevant window is the one closest to assessment. Childhood injury includes violent shaking, consistent with the treatment of partner-inflicted shaking, so that the same definition applies on both sides of the model. Non-partner and childhood injury counts were log-transformed and defined identically to the primary exposure, including violent shaking.

Individually, these measures showed weak associations with cognitive measures, including psychomotor speed: childhood maltreatment ($r = -0.17$, $P = 0.017$), post-traumatic stress ($r = -0.15$, $P = 0.041$), depression ($r = -0.14$, $P = 0.051$), abuse severity ($r = -0.13$, $P = 0.060$), anxiety ($r = -0.12$, $P = 0.101$), and alcohol use ($r = -0.04$, $P = 0.594$). None was of a magnitude that could account for the association with injury count, which was $r = -0.24$ before any of them was entered and −0.22 with all of them adjusted (Table S8). In addition, only 10 women were in a current abusive relationship at assessment, and adjusting for a current abusive relationship did not affect the association, so the cognitive deficit does not appear to reflect ongoing threat.

Reporting

This observational study is reported in accordance with the STROBE statement for cross-sectional studies [95]. A completed STROBE checklist is provided with the submission. The study was approved by the institutional review board of Mass General Brigham, and all participants gave informed consent.

Statistical analysis

The study addressed whether the number of partner-inflicted brain injuries a woman sustained predicts her cognitive performance. The primary outcome was cognitive performance across the sixteen domains the battery reports, specified as a set rather than as a single index because the question concerns cognition broadly and no one domain was expected *a priori* to carry the association. We report each association with its confidence interval, and all tests were two-sided at an alpha of 0.05.

The primary exposure was the natural log of injury count, winsorized at 25. For each of the sixteen cognitive domains, we fitted a linear regression with the domain score as the outcome and injury count and the eleven covariates as predictors. The coefficient on injury count was tested by Wald test and is reported as a partial correlation, so that effects are comparable across domains. Six of the sixteen reached $P < 0.05$, against 0.8 expected were no association present.

medRxiv preprint doi: https://doi.org/10.64898/2026.09.24.26363984; this version posted September 27, 2026. The copyright holder for this preprint (which was not certified by peer review) is the author/funder, who has granted medRxiv a license to display the preprint in perpetuity.
It is made available under a CC-BY 4.0 International license .

Under Benjamini-Hochberg control across the sixteen domains, four associations remain significant at $q < 0.05$ (psychomotor speed, reaction time, processing speed and motor speed). Cognitive age was derived separately for each domain that reports a raw score, and is presented for those showing a significant association with injury count. Analyses use complete cases, and the number of observations contributing to each estimate is reported with that estimate. All remaining analyses are secondary or sensitivity analyses.

The sixteen domain associations are reported as effect estimates with confidence intervals and unadjusted P values, and interpreted as one profile rather than as sixteen separate tests; six reached $P < 0.05$ against 0.8 expected were no association present, with no domain treated as established or excluded on the basis of a threshold alone [96]. Comparisons of domain means against population norms were corrected by Bonferroni across the sixteen domains. Secondary analyses, including the comparison repeated at each cut-off from one to twelve injuries, alternative handling of extreme counts, alternative covariate sets, and restriction to mild BIs only, are reported as effect estimates with confidence intervals and unadjusted P values. These secondary analyses re-estimate a single contrast under alternative analytic choices rather than testing new hypotheses.

Brain injury count was severely right-skewed (median 1, interquartile range 0 to 4, range 0 to 1,095; adjusted Fisher-Pearson $G1 = 11.0$) and is therefore summarized by median and interquartile range and modeled as log(1 + count) after capping at 25. Among plausible exposure codings, every alternative except the binary any-injury contrast fitted the data comparably by Akaike information criterion, the logarithmic specification falling within 0.24 units of the best-fitting coding (Fig. S1). As the functional form was selected empirically, we tested whether the inference depended on it using prespecified exposure bands, uncapped and untransformed counts, ordinal injury-frequency ratings, and rank-based analyses; the association was consistent across specifications, and the uncapped and untransformed alternatives are reported in Table S5.

Cognitive standard scores were approximately normally distributed (global cognition, Shapiro-Wilk $W = 0.98$) and are summarized by mean and standard deviation, in standard-score units with a normative mean of 100 and standard deviation of 15. Cognitive age gaps are reported in years. Group comparisons used Welch unpaired t tests, which do not assume equal variances, with Cohen's d and 95% confidence intervals. Cohen's d is reported unsigned throughout, with the direction of each difference given by the group means. Comparisons against population norms used one-sample t-tests against a hypothetical mean of 100. Effect sizes are referenced to the distribution each comparison is made against. For comparisons with population norms, d is the deviation of the group mean from 100 divided by the normative standard deviation of 15, so that it reads directly as a position in the normative distribution. For comparisons between groups within the cohort, d is the difference in means divided by the pooled standard deviation of the two groups compared. Sample sizes and t statistics are reported for every domain and every group in Tables S2 and S3 for improved replicability.

Trend across ordered exposure bands was tested by linear contrast. Bootstrap procedures used 4,000 to 10,000 resamples with a fixed seed. No data were imputed. Analyses use complete cases, and the number of observations contributing to each estimate is stated with that estimate; coverage ranges from 169 of 207 for global cognition, which requires every component subtest to be valid, to 206 for reasoning, and 206 of 207 women are complete on all eleven covariates. Sources of missing cognitive data, and the consequences of the exclusion rule that generates most of it, are examined directly in the Results and in Fig. 5.

medRxiv preprint doi: https://doi.org/10.64898/2026.09.24.26363984; this version posted September 27, 2026. The copyright holder for this preprint (which was not certified by peer review) is the author/funder, who has granted medRxiv a license to display the preprint in perpetuity.

It is made available under a CC-BY 4.0 International license .

Analyses were conducted in Python 3.12 and GraphPad Prism 11.0.2. The deposited dataset is provided as Data S1. Sample size was fixed by the size of the cohort and was not determined by power calculation. With 63 uninjured women and 32 reporting ten or more injuries, the smallest within-cohort difference detectable at 80% power is $d = 0.61$. Where a comparison between injury groups on a given cognitive domain does not reach significance, we describe it as no difference detected rather than as no difference, and we report confidence intervals so that readers can see how large a difference remains possible.

medRxiv preprint doi: https://doi.org/10.64898/2026.09.24.26363984; this version posted September 27, 2026. The copyright holder for this preprint (which was not certified by peer review) is the author/funder, who has granted medRxiv a license to display the preprint in perpetuity.

It is made available under a CC-BY 4.0 International license .

Supplementary Text

Robustness to analytic choices

We tested whether the association was robust to alternative analytic decisions and potential sources of bias, including the influence of individual participants and adjustment for additional covariates.

Our primary analysis winsorized partner-inflicted BI counts at 25, following prior work in this cohort [42], as well as log-transformed to address extreme right-skewness (adjusted Fisher-Pearson $g_1 = 11.0$). This was done to prevent extreme counts from disproportionately influencing continuous analyses. Consequently, fifteen women who reported more than 25 injuries, with counts reaching 1,095, all were assigned a value of 25 in the primary continuous analysis. To test whether this decision to cap the exposure affected the association, we repeated the analysis using the full reported counts. The association was slightly stronger when counts were left uncapped (partial $r = -0.24$, $P = 0.0009$). As a separate robustness analysis, we then excluded the fifteen women who exceeded the cap entirely. The association again remained essentially unchanged (Table S5).

Relatedly, to test whether the association was driven by a small number of influential participants, we examined Cook's distance and standardised DFBETAs for the group coefficient in a model of cognitive age gap on ten-or-more versus no injury. No single participant exerted significant influence (maximum Cook's D = 0.083; maximum absolute standardised DFBETA = 0.331). The corresponding values for the exposure coefficient of the primary adjusted model were 0.067 and 0.358.

Our primary analysis adjusted for eleven covariates. Two further covariates were tested in the subsample for whom they were recorded. Adding both covariates minimally strengthened the association (partial $r = -0.27$, $P = 0.071$ when excluded; partial $r = -0.30$, $P = 0.046$ when included). Neither neurological disorder ($r = -0.001$) nor current cannabis use ($r = 0.073$) was associated with injury count, so neither can account for the result. These covariates were not added to the primary model because they are recorded for less than a third of the sample, and doing so would have cost the majority of the observations to adjust for variables that do not confound the exposure.

Finally, our primary analysis grouped women into none, one to three, four to nine, and ten or more partner-inflicted BIs. To test whether the observed dose-response relationship between injury burden and cognitive performance depended on these cut-points, we repeated the analysis using four alternative grouping schemes (e.g., one to two rather than one to three; seven or more rather than ten or more as the highest-exposure band), varying the lower and upper boundaries and, in one analysis, dividing exposure into five bands. Across all four specifications, the gradient remained similar between injury exposure bands and cognitive age gaps (P for trend = 0.005 to 0.022). Treating injury count continuously rather than grouping it likewise preserved the association (Spearman $\rho = 0.14$, $P = 0.043$).

Mechanism of injury

Splitting the cohort by mechanism, psychomotor speed was 93.5 in women with blunt-force injury only, 95.1 in women with strangulation-related injury only, and 88.8 in women with both, against 95.8 in women with neither. Women with blunt-force versus strangulation-related injury alone did not differ from one another ($d = 0.08$, $P = 0.69$), and no other cognitive domain distinguished these two groups. When blunt-force and strangulation injury counts were entered simultaneously, greater

medRxiv preprint doi: https://doi.org/10.64898/2026.09.24.26363984; this version posted September 27, 2026. The copyright holder for this preprint (which was not certified by peer review) is the author/funder, who has granted medRxiv a license to display the preprint in perpetuity.

It is made available under a CC-BY 4.0 International license .

blunt-force injury burden remained associated with poorer psychomotor speed (partial $r = –0.20$, $P = 0.008$), whereas strangulation-related injury burden did not (partial $r = −0.05$, $P = 0.52$). Similarly, psychomotor speed declined across increasing bands of blunt-force injury count (P for trend = 0.018), but not across bands of strangulation-related injury count ($P = 0.25$). These comparisons were substantially better powered for blunt-force injury, however, because only 16 women had strangulation-related injury without any blunt-force injury, so the failure to resolve a strangulation effect here should not be read as evidence that strangulation is cognitively harmless. Notably, women exposed to both mechanisms performed worst, suggesting that cumulative injury burden may be more important than injury mechanism alone.

medRxiv preprint doi: https://doi.org/10.64898/2026.09.24.26363984; this version posted September 27, 2026. The copyright holder for this preprint (which was not certified by peer review) is the author/funder, who has granted medRxiv a license to display the preprint in perpetuity.

It is made available under a CC-BY 4.0 International license .

1074 ## Supplementary Figures

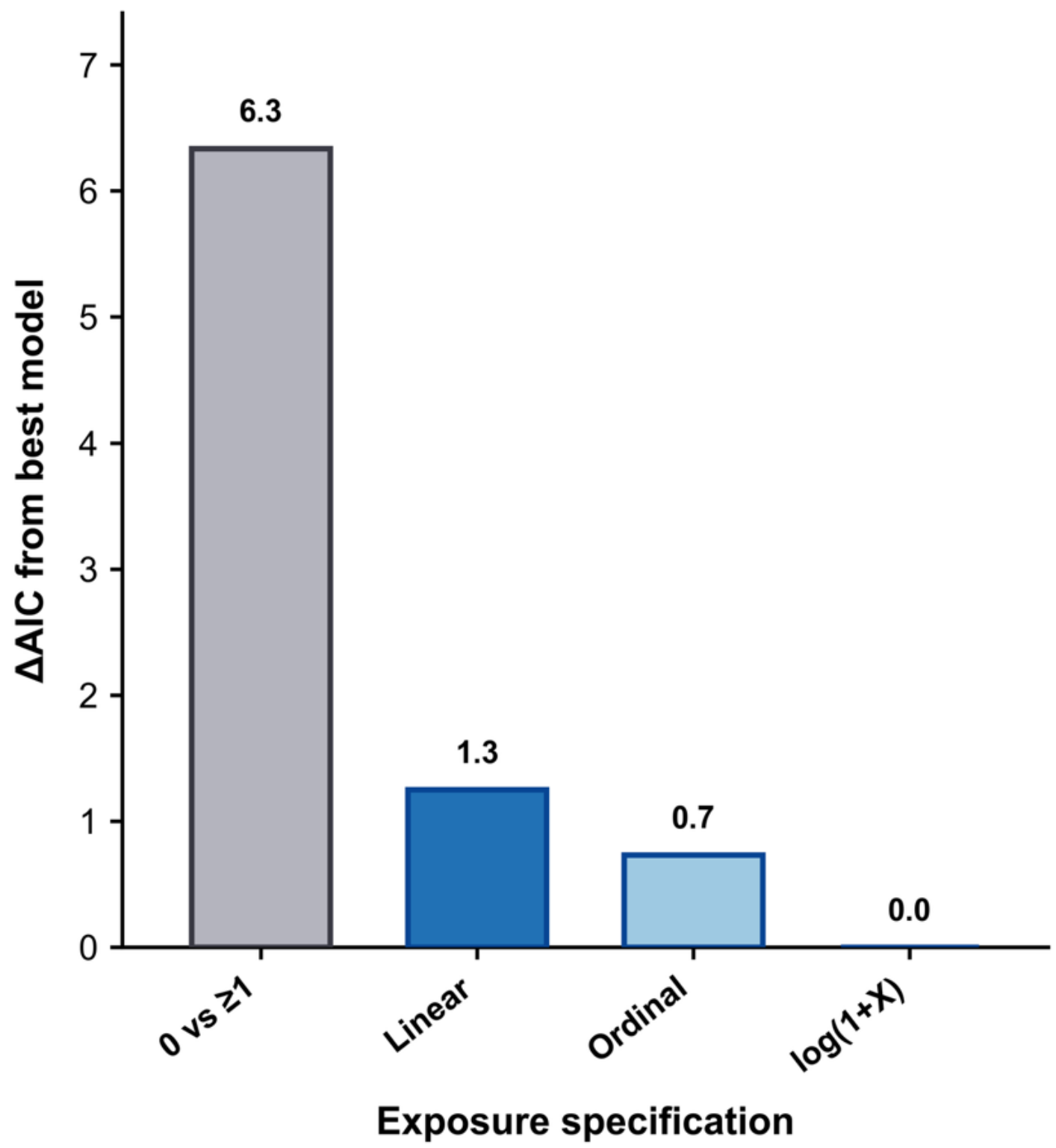

Fig. S1. Model fit under alternative codings of injury exposure. Bars give the difference in Akaike information criterion (AIC) between each coding of partner-inflicted injury and the best-fitting coding, in age-adjusted models of psychomotor speed ($n = 197$). Lower AIC indicates better fit. Injury counts are capped at 25, as in the primary analysis. The logarithmic coding fits best (AIC = 1144.1), followed closely by the ordinal bands (1144.8) and the linear count (1145.3). Notably, the binary any-injury specification, the approach most often used in this literature, fits worst (1150.4). Leaving the counts uncapped does not change this. The logarithmic coding was therefore used, and the sensitivity analyses in Tables S4 to S7 show the association does not depend on this choice.

medRxiv preprint doi: https://doi.org/10.64898/2026.09.24.26363984; this version posted September 27, 2026. The copyright holder for this preprint (which was not certified by peer review) is the author/funder, who has granted medRxiv a license to display the preprint in perpetuity.

It is made available under a CC-BY 4.0 International license .

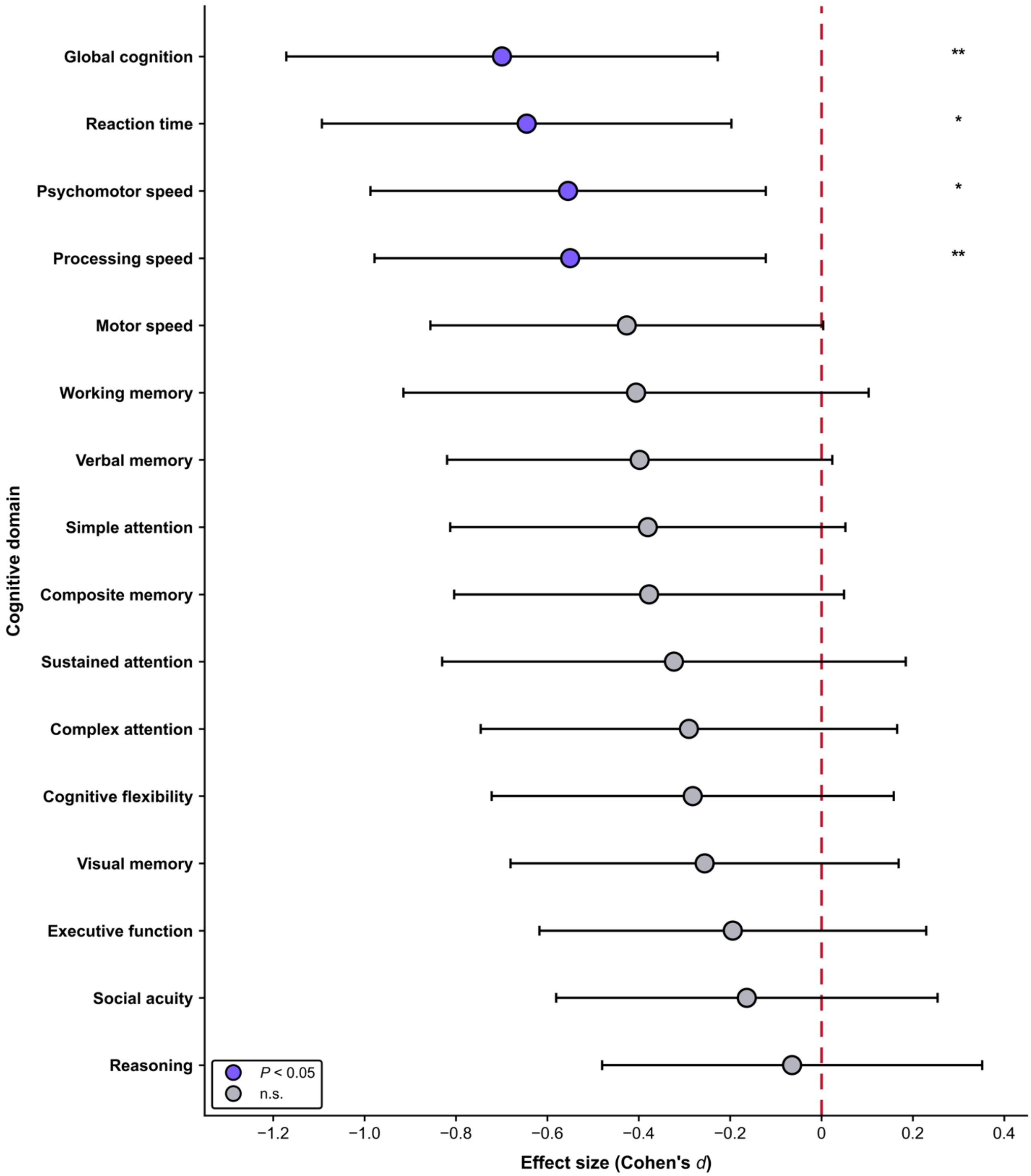

Fig. S2. All sixteen cognitive domains ranked by effect size. Standardized difference between women reporting ten or more partner-inflicted brain injuries and women reporting none, with 95% confidence intervals. Global cognition shows the largest difference, followed by reaction time, psychomotor speed and processing speed ($P < 0.05$). The remaining twelve cognitive domains do not.

medRxiv preprint doi: https://doi.org/10.64898/2026.09.24.26363984; this version posted September 27, 2026. The copyright holder for this preprint (which was not certified by peer review) is the author/funder, who has granted medRxiv a license to display the preprint in perpetuity.

It is made available under a CC-BY 4.0 International license .

Supplementary Tables

Table S1. Sample characteristics. Values are mean (SD) unless stated. *P* values compare women reporting ten or more partner-inflicted brain injuries against women reporting none, by Welch unpaired t-test. Group sizes in the header apply to the demographic, injury and psychiatric rows; each cognition row rests on a smaller complete-case sample, reported per domain in Table S3. Standard scores have a population mean of 100 and standard deviation of 15.

**Characteristic**	**All (*n* = 207)**	**No injury (*n* = 68)**	**10 or more (*n* = 34)**	***P***
**Demographics**				
Age, years	41.0 (13.2)	38.9 (14.5)	40.6 (11.2)	0.532
Education, years	15.7 (2.8)	16.7 (2.6)	15.6 (3.1)	0.071
Non-White, %	30	28	29	1.000
Hispanic, %	16	19	18	1.000
**Partner-inflicted brain injury**				
Any injury, %	67	0	100	
Repetitive injury, %	42	0	100	
Strangulation-related, %	27	0	53	
Exceeding the 25-event cap, %	7	0	44	
Injuries, median (IQR)	1 (0 to 4)	0 (0 to 0)	22 (12 to 40)	
**Psychiatric and abuse measures**				
Post-traumatic stress (PCL-5)	33.5 (19.3)	30.2 (18.0)	41.9 (16.4)	0.002
Depression (PHQ-9)	9.6 (5.8)	8.5 (5.7)	11.0 (5.2)	0.033
Anxiety (GAD-7)	8.3 (5.7)	7.7 (5.2)	10.4 (5.6)	0.019
Alcohol use (AUDIT)	3.4 (5.2)	2.8 (3.3)	3.3 (4.8)	0.606
Childhood maltreatment (CTQ)	58.3 (22.1)	53.8 (21.8)	64.9 (20.0)	0.013
Abuse severity (CAS)	6.9 (11.1)	6.5 (10.9)	10.6 (15.1)	0.165
Currently with an abusive partner (*n*)	10	4	2	
**Cognition, standard score**				
Global cognition	93.8 (11.4)	95.0 (9.7)	87.6 (11.7)	0.007
Psychomotor speed	93.5 (18.3)	95.8 (17.0)	86.2 (17.9)	0.015
Reaction time	88.9 (18.0)	90.9 (14.9)	79.6 (21.8)	0.014
Processing speed	97.7 (15.6)	100.3 (17.7)	91.2 (13.7)	0.006
Motor speed	93.2 (17.6)	94.8 (14.9)	88.0 (17.9)	0.070
Composite memory	92.1 (18.4)	93.9 (17.6)	86.9 (20.7)	0.104
Verbal memory	92.6 (18.4)	95.0 (18.3)	87.8 (17.8)	0.064
Visual memory	94.6 (16.4)	95.3 (15.7)	90.8 (20.4)	0.284
Complex attention	96.9 (17.1)	97.0 (16.0)	91.9 (20.2)	0.253
Cognitive flexibility	92.0 (16.6)	91.7 (15.2)	87.3 (16.4)	0.224
Executive function	91.7 (16.7)	90.3 (16.4)	87.2 (16.0)	0.368
Social acuity	99.8 (16.5)	101.2 (15.4)	98.8 (12.5)	0.412
Reasoning	95.1 (16.0)	94.9 (15.6)	93.9 (15.6)	0.763
Working memory	102.9 (11.8)	105.0 (11.6)	100.4 (10.8)	0.113
Sustained attention	104.2 (9.5)	105.5 (8.1)	102.8 (9.4)	0.245
Simple attention	90.9 (21.7)	93.7 (20.1)	85.7 (22.7)	0.102
Lost ≥1 score to validity criteria, %	40	43	38	—

medRxiv preprint doi: https://doi.org/10.64898/2026.09.24.26363984; this version posted September 27, 2026. The copyright holder for this preprint (which was not certified by peer review) is the author/funder, who has granted medRxiv a license to display the preprint in perpetuity.

It is made available under a CC-BY 4.0 International license .

Table S2. All sixteen cognitive domains against population norms across whole cohort and among those with history of partner-inflicted brain injury. One-sample *t* tests against a normative mean of 100, with Bonferroni correction across sixteen tests. Sample sizes vary across domains because scores flagged as invalid by the battery's validity indicator were excluded, leaving 143 to 206 of the 207 women per domain. Global cognition additionally requires every component subtest to be valid, therefore resulting in a lower sample size.

	Whole cohort				≥ 1 partner-inflicted BI				≥ 10 partner-inflicted BIs			
**Domain**	**n**	**Mean**	**t**	$P_{Bonferroni}$	**n**	**Mean**	**t**	$P_{Bonferroni}$	**n**	**Mean**	**t**	$P_{Bonferroni}$
Reaction time	190	88.9	−8.48	<0.0001	130	88.0	−7.10	<0.0001	30	79.6	−5.12	0.0003
Simple attention	192	90.9	−5.82	<0.0001	128	89.5	−5.31	<0.0001	31	85.7	−3.51	0.0232
Executive function	199	91.7	−7.03	<0.0001	133	92.4	−5.23	<0.0001	32	87.2	−4.53	0.0013
Cognitive flexibility	188	92.0	−6.62	<0.0001	128	92.1	−5.15	<0.0001	30	87.3	−4.25	0.0032
Composite memory	201	92.1	−6.11	<0.0001	136	91.2	−5.48	<0.0001	32	86.9	−3.59	0.0182
Verbal memory	204	92.6	−5.71	<0.0001	138	91.5	−5.44	<0.0001	33	87.8	−3.92	0.0069
Motor speed	197	93.2	−5.42	<0.0001	134	92.5	−4.65	0.0001	32	88.0	−3.79	0.0103
Psychomotor speed	197	93.5	−5.02	<0.0001	134	92.4	−4.71	<0.0001	32	86.2	−4.36	0.0021
Global cognition	169	93.8	−7.10	<0.0001	117	93.2	−6.05	<0.0001	28	87.6	−5.58	0.0001
Visual memory	201	94.6	−4.72	<0.0001	136	94.2	−4.04	0.0014	32	90.8	−2.54	0.2585
Reasoning	206	95.1	−4.39	0.0003	138	95.2	−3.48	0.0109	33	93.9	−2.24	0.5179
Complex attention	174	96.9	−2.41	0.2690	118	96.8	−1.95	0.8560	28	91.9	−2.12	0.6945
Processing speed	204	97.7	−2.08	0.6198	137	96.5	−2.88	0.0747	32	91.2	−3.63	0.0162
Social acuity	201	99.8	−0.14	1.0000	134	99.1	−0.58	1.0000	33	98.8	−0.53	1.0000
Working memory	143	102.9	2.96	0.0584	95	101.9	1.55	1.0000	22	100.4	0.16	1.0000
Sustained attention	143	104.2	5.31	<0.0001	95	103.5	3.42	0.0147	22	102.8	1.38	1.0000

Table S3. Standardized differences across all sixteen cognitive domains for women reporting ten or more partner-inflicted brain injuries versus women reporting none. *P* values are unadjusted.

**Domain**	***n* (no /10+)**	**No injury**	**Ten or more**	***d***	**95% CI**	***P***
Global cognition	52/28	95.0	87.6	0.70	0.23, 1.17	0.007
Reaction time	60/30	90.9	79.6	0.64	0.20, 1.09	0.014
Psychomotor speed	63/32	95.8	86.2	0.55	0.12, 0.99	0.015
Processing speed	67/32	100.3	91.2	0.55	0.12, 0.98	0.006
Motor speed	63/32	94.8	88.0	0.43	0.00, 0.86	0.070
Working memory	48/22	105.0	100.4	0.41	-0.10, 0.91	0.113
Verbal memory	66/33	95.0	87.8	0.40	-0.02, 0.82	0.064
Composite memory	65/32	93.9	86.9	0.38	-0.05, 0.80	0.104
Simple attention	64/31	93.7	85.7	0.38	-0.05, 0.81	0.102
Sustained attention	48/22	105.5	102.8	0.32	-0.18, 0.83	0.245
Complex attention	56/28	97.0	91.9	0.29	-0.17, 0.75	0.253

medRxiv preprint doi: https://doi.org/10.64898/2026.09.24.26363984; this version posted September 27, 2026. The copyright holder for this preprint (which was not certified by peer review) is the author/funder, who has granted medRxiv a license to display the preprint in perpetuity.

It is made available under a CC-BY 4.0 International license .

**Domain**	***n* (no /10+)**	**No injury**	**Ten or more**	***d***	**95% CI**	***P***
Cognitive flexibility	60/30	91.7	87.3	0.28	-0.16, 0.72	0.224
Visual memory	65/32	95.3	90.8	0.26	-0.17, 0.68	0.284
Executive function	66/32	90.3	87.2	0.19	-0.23, 0.62	0.368
Social acuity	67/33	101.2	98.8	0.16	-0.25, 0.58	0.412
Reasoning	68/33	94.9	93.9	0.06	-0.35, 0.48	0.763

Table S4. Sensitivity of the effect to the injury count used to define high exposure. Twelve thresholds were tested, with psychomotor speed as the outcome. Each threshold is compared against women reporting no partner-inflicted brain injury.

**Threshold defining high exposure**	***n exposed***	***d***	**95% CI**	***P***
1 or more	134	0.19	-0.11, 0.49	0.200
2 or more	83	0.36	0.03, 0.69	0.032
3 or more	65	0.39	0.04, 0.74	0.028
4 or more	54	0.46	0.09, 0.83	0.015
5 or more	45	0.61	0.22, 1.00	0.003
6 or more	40	0.53	0.13, 0.94	0.012
7 or more	39	0.53	0.13, 0.94	0.012
8 or more	37	0.50	0.09, 0.91	0.022
9 or more	34	0.48	0.06, 0.90	0.032
10 or more	32	0.55	0.12, 0.99	0.015
11 or more	28	0.57	0.12, 1.03	0.019
12 or more	26	0.63	0.16, 1.10	0.014

Table S5. Sensitivity to the handling of extreme injury counts. The primary analysis caps counts at 25; alternatives exclude women above the stated value. Gap is the mean cognitive age gap for women reporting ten or more injuries against the general population, bounded to ages 18 to 90 as in the main analyses; d is the standardized difference in psychomotor speed against women reporting none. Every alternative preserves the direction of the effect, with gaps ranging from 19.0 to 24.6 years and d from 0.45 to 0.67 against 0.55 in the primary analysis.

**Handling of extreme counts**	***n***	**Gap (years)**	***d***	**95% CI**
Capped at 25 (primary)	207	20.8	0.55	0.12 to 0.99
Exclude counts above 145	202	19.1	0.45	0.00 to 0.91
Exclude counts above 100	202	19.1	0.45	0.00 to 0.91
Exclude counts above 50	201	19.0	0.45	-0.01 to 0.91
Exclude counts above 25	192	24.6	0.67	0.13 to 1.22
Exclude counts above 16	188	23.3	0.64	0.05 to 1.22

Table S6. Sensitivity to the covariate set. Partial correlations between log injury count and psychomotor speed under progressively fuller adjustment.

**Covariate set**	***n***	***Partial r***	***P***
+ Age	197	-0.198	0.0055
+ Education	197	-0.184	0.0099
+ Race	197	-0.182	0.0112
+ Non-partner adult brain injury	197	-0.240	0.0008
+ Childhood brain injury	197	-0.231	0.0012
+ Post-traumatic stress	197	-0.215	0.0028

medRxiv preprint doi: https://doi.org/10.64898/2026.09.24.26363984; this version posted September 27, 2026. The copyright holder for this preprint (which was not certified by peer review) is the author/funder, who has granted medRxiv a license to display the preprint in perpetuity.

It is made available under a CC-BY 4.0 International license .

**Covariate set**	***n***	***Partial r***	***P***
+ Depression	197	-0.222	0.0021
+ Anxiety	197	-0.223	0.0020
+ Alcohol use	196	-0.232	0.0014
+ Childhood maltreatment	196	-0.231	0.0015
+ Abuse severity	196	-0.221	0.0025

Table S7. Sensitivity to the injury definition. The primary definition counts all qualifying partner-inflicted brain injuries, including moderate-to-severe events, whereas the alternative definition counts only mild injuries. Under the mild-only definition, women whose injuries were exclusively moderate-to-severe are therefore classified as having no qualifying injury. Moderate-to-severe events are rare in this cohort, with nine events among eight women, and only one woman is reclassified from the exposed group to the reference group. That woman contributes no valid cognitive score, so every estimate below is identical under the two definitions, and this sensitivity analysis has little power to distinguish them. Gaps are bounded to ages 18 to 90 as in the primary analyses.

**Injury definition**	**n (0 / 10+)**	**Absolute gap**	**vs uninjured**	***d***
Total brain injury (primary)	63/32	20.8	11.3	0.55
Mild brain injury only	63/32	20.8	11.3	0.55

Table S8. Alternative explanations tested directly. Each measure is related both to cognition and to injury count, adjusted for age. A confounder would have to predict both. Childhood maltreatment and post-traumatic stress are each weakly associated with cognition, but neither is of a magnitude that could account for the association with injury count.

**Measure**	***n***	**r with cognition**	***P***	**r with injury count**	***P***
Post-traumatic stress	197	-0.146	0.041	0.213	0.003
Depression	197	-0.140	0.051	0.121	0.090
Anxiety	197	-0.120	0.093	0.149	0.037
Alcohol use	196	-0.040	0.583	0.048	0.502
Childhood maltreatment	197	-0.171	0.017	0.134	0.061
Abuse severity	197	-0.135	0.059	0.172	0.016

Table S9. Cognitive domains and the subtests from which each is scored. Descriptions are provided verbatim from the CNS Vital Signs score report.

<table>
<tr><th>Cognitive test</th><th>Derived CNSVS domain</th><th>CNSVS test description</th></tr>
<tr><td rowspan="2">Verbal Memory Test (VBM)</td><td>Composite Memory</td><td rowspan="2">Fifteen target words are presented and must then be picked out from among fifteen distractors, with the recognition trial repeated at the end of the battery. The score is the number of target words correctly identified, indexing recognition and retrieval of verbal material; lower scores indicate weaker verbal memory.</td></tr>
<tr><td>Verbal Memory</td></tr>
<tr><td rowspan="2">Visual Memory Test (VSM)</td><td>Composite Memory</td><td rowspan="2">Fifteen geometric figures are presented and must then be picked out from among fifteen distractors, with the recognition trial repeated at the end of the battery. The score is the number of target figures correctly identified, indexing recognition and retrieval of visual material; lower scores indicate weaker visual memory.</td></tr>
<tr><td>Visual Memory</td></tr>
</table>

medRxiv preprint doi: https://doi.org/10.64898/2026.09.24.26363984; this version posted September 27, 2026. The copyright holder for this preprint (which was not certified by peer review) is the author/funder, who has granted medRxiv a license to display the preprint in perpetuity.

It is made available under a CC-BY 4.0 International license .

Cognitive test	Derived CNSVS domain	CNSVS test description
**Finger Tapping Test (FTT)**	Psychomotor Speed<br>Motor Speed	Three timed tapping trials are completed with each hand. The score is the number of taps per hand, indexing fine motor speed and control; fewer taps indicate motor slowing, and rates differ systematically between the dominant and non-dominant hand.
**Symbol Digit Coding (SDC)**	Processing Speed<br>Psychomotor Speed	Digits are matched to symbols against the clock. Performance depends jointly on visual scanning, perception, short-term visual memory and motor output, so the score indexes processing speed broadly rather than any single component; errors may arise from impulsive responding as well as from misperception.
**Stroop Test (ST)**	Reaction Time<br>Complex Attention	Simple and complex reaction times are recorded across congruent and incongruent colour-word conditions, indexing response inhibition and the capacity to keep pace as the instructions change and grow more demanding. Longer latencies indicate slowed processing.
**Shifting Attention Test (SAT)**	Cognitive Flexibility<br>Executive Function<br>Complex Attention	Matching rules change without warning, so responses must be re-mapped while competing rules are held in mind. The score combines correct responses, errors and response latency, and indexes set-shifting and the handling of concurrent task demands.
**Continuous Performance Test (CPT)**	Simple Attention<br>Complex Attention	Responses are made to a designated target over a sustained interval, yielding measures of vigilance and choice reaction time. Unimpaired performance is close to ceiling, so a small number of errors, or unusually long latencies, carry diagnostic weight.
**Perception of Emotions Test (POET)**	Social Acuity	Facial expressions are classified as happy, calm, angry or sad, indexing the perception and identification of emotion. Response latencies are considerably longer than on the other subtests, reflecting the additional processing that emotion recognition requires.
**Non-Verbal Reasoning Test (NVRT)**	Reasoning	Fifteen matrix problems of increasing difficulty are presented, each for 14.5 seconds, indexing the perception of visual-abstract information and the recognition of relationships among its elements.
**Four-Part Continuous Performance Test (FPCPT)**	Working Memory<br>Sustained Attention	Four parts of increasing load: a simple reaction-time block; a continuous-performance discrimination block yielding choice reaction times; a one-back block; and a two-back block. The two-back block yields the working memory score, and the second, third and fourth blocks together yield sustained attention.

Table S10. Association between injury count and each speed and memory domain, adjusted for age. Speed domains vary with injury count more strongly than memory domains, the reverse of the pattern memory-driven under-reporting would produce. Averaged across domains, the age-adjusted association is larger for speed (0.19) than for memory (0.12). The two sets share participants, so we report the contrast descriptively rather than testing it.

medRxiv preprint doi: https://doi.org/10.64898/2026.09.24.26363984; this version posted September 27, 2026. The copyright holder for this preprint (which was not certified by peer review) is the author/funder, who has granted medRxiv a license to display the preprint in perpetuity.

It is made available under a CC-BY 4.0 International license .

Domain type	Domain	*n*	|*r*| with injury count	*P*
Speed	Psychomotor speed	197	0.198	0.005
Speed	Motor speed	197	0.151	0.035
Speed	Processing speed	204	0.202	0.004
Speed	Reaction time	190	0.210	0.004
Memory	Composite memory	201	0.131	0.064
Memory	Verbal memory	204	0.138	0.050
Memory	Visual memory	201	0.095	0.183
	Speed mean		0.190	
	Memory mean		0.121	

Table S11. Cognitive association by injury mechanism. Partial correlations between psychomotor speed and the log count of each injury mechanism, adjusted for the eleven covariates of the primary model, entered first separately and then together. Total injury count is not entered, because blunt-force events constitute 89% of all injuries and the two are collinear (r = 0.998 for the raw counts, and 0.884 for the log-transformed predictors actually entered), so a model containing both is uninterpretable. Strangulation injuries were reported by 56 women with a median of two, so this comparison is better powered to detect an effect of blunt-force injury.

Injury mechanism	*n*	*Partial r*	*P*
Blunt-force injuries, entered alone	196	−0.208	0.0046
Strangulation injuries, entered alone	196	−0.086	0.2457
Blunt-force injuries, adjusted for strangulation	196	−0.195	0.0079
Strangulation injuries, adjusted for blunt force	196	−0.047	0.5220

Table S12. Cognitive age gap by domain. Cognitive age was derived separately for each domain by regressing the raw score on chronological age and the age-adjusted standard score, converting the standard-score deficit into years, and bounding the resulting cognitive age to 18 to 90 years. Values give the gap for women reporting ten or more partner-inflicted brain injuries, against the general population and against women in this cohort reporting none. The years-per-point column gives the conversion factor, which is the number of years of age equivalent to one standard-score point in that domain. Conversion factors vary widely because domains differ in how steeply their raw scores decline with age, and estimates from domains with shallow slopes, such as simple attention and social acuity, are correspondingly less precise. Two-sided t tests, unadjusted. Cognitive age was derived for each of the fifteen domains that report a raw score. Given that global cognition is an average of age-standardized scores across multiple cognitive domains, and therefore has no raw performance measure that changes with age, a cognitive age for global cognition cannot be calculated.

Domain	Years per point	*n*	Gap vs population (95% CI)	*P*	Gap within cohort (95% CI)	*P*
**Simple attention**	32.79	31	28.1 (14.9 to 41.4)	0.0002	14.2 (-1.1 to 29.5)	0.074
**Reaction time**	2.04	30	27.8 (18.1 to 37.4)	<0.0001	11.9 (1.1 to 22.7)	0.036
**Verbal memory**	5.31	33	26.0 (15.8 to 36.2)	<0.0001	13.7 (1.2 to 26.3)	0.036
**Motor speed**	4.50	32	24.6 (13.6 to 35.6)	<0.0001	6.0 (-6.6 to 18.5)	0.353
**Composite memory**	4.14	32	22.7 (12.4 to 33.0)	<0.0001	6.2 (-6.2 to 18.7)	0.331
**Cognitive flexibility**	2.24	30	22.6 (13.0 to 32.1)	<0.0001	8.0 (-2.9 to 18.9)	0.158
**Executive function**	2.19	32	21.9 (13.3 to 30.6)	<0.0001	6.3 (-3.8 to 16.5)	0.227

medRxiv preprint doi: https://doi.org/10.64898/2026.09.24.26363984; this version posted September 27, 2026. The copyright holder for this preprint (which was not certified by peer review) is the author/funder, who has granted medRxiv a license to display the preprint in perpetuity.

It is made available under a CC-BY 4.0 International license .

**Psychomotor speed**	1.77	32	20.8 (11.6 to 29.9)	<0.0001	11.3 (1.2 to 21.5)	0.033
**Visual memory**	4.57	32	19.7 (8.4 to 31.1)	0.0013	1.7 (-11.7 to 15.1)	0.807
**Reasoning**	5.65	33	15.9 (5.8 to 25.9)	0.0030	1.7 (-10.1 to 13.5)	0.783
**Complex attention**	4.41	28	11.4 (-2.5 to 25.3)	0.1033	2.2 (-13.2 to 17.7)	0.779
**Processing speed**	1.30	32	11.4 (5.0 to 17.8)	0.0010	8.6 (1.3 to 15.8)	0.024
**Social acuity**	25.46	33	11.2 (-3.2 to 25.6)	0.1220	3.9 (-12.6 to 20.4)	0.644
**Working memory**	6.31	22	5.0 (-7.8 to 17.8)	0.4277	4.6 (-10.0 to 19.1)	0.542
**Sustained attention**	6.45	22	-6.8 (-17.3 to 3.6)	0.1883	-2.2 (-14.3 to 9.8)	0.717
